# Supplementary material for: Investigation of Inflammation in Lewy Body Dementia: A Systematic Scoping Review
Source: Int J Mol Sci. 2023 Jul 28;24(15):12116. doi: 10.3390/ijms241512116 (PMC10418754; doi:10.3390/ijms241512116)
Supplement: Supplementary file 1 [file ijms-24-12116-s001.zip › Figure S2 Example of full search strategy.pdf]

Figure S2: Example of full search strategy

Database:	Ovid MEDLINE(R) and Epub Ahead of Print, In-Process, In-Data-Review & Other Non-Indexed Citations and Daily <1946 to January 26, 2023>	
Search strategy:	1	Lewy.mp.
	2	dementia with lewy bodies.mp.
	3	microglia.mp.
	4	inflammat*.mp.
	5	Neurogenic Inflammation/
	6	neuroinflammat*.mp. [mp=title, abstract, original title, name of substance word, subject heading word, floating sub-heading word, keyword heading word, organism supplementary concept word, protocol supplementary concept word, rare disease supplementary concept word, unique identifier, synonyms]
	7	parkinsons disease dementia.mp.
	8	dementia.mp.
	9	Parkinson Disease/
	10	parkinsons disease.mp.
	11	9 or 10
	12	8 and 11
	13	1 and 8
	14	2 or 7 or 12 or 13
	15	3 or 4 or 5 or 6
	16	14 and 15
