# Supplementary material for: Investigation of Inflammation in Lewy Body Dementia: A Systematic Scoping Review
Source: Int J Mol Sci. 2023 Jul 28;24(15):12116. doi: 10.3390/ijms241512116 (PMC10418754; doi:10.3390/ijms241512116)
Supplement: Supplementary file 1 [file ijms-24-12116-s001.zip › Tables S1-S5 Summaries of included studies.pdf]

Supplementary material - Summaries of included studies

Contents

Notes 1

Table S1. Postmortem studies 2

Table S2. Imaging 12

Table S3. Cerebrospinal fluid 14

Table S4. Blood 21

Table S5. Genetic factors 29

Abbreviations 31

References 33

Notes

- *Indicates studies that are repeated across multiple tables, due to analysis of multiple substrates (e.g., CSF and blood). The contents are identical between tables.
- Only markers of inflammation assessed in Lewy body dementia (LBD) cases are reported.
- A range of sample sizes is provided (e.g., DLB 8-9) if different components of the study contained groups of different sizes.
- In some earlier studies, cases described as having Parkinson's disease and dementia without clear application of the '1 year rule' criteria to distinguish dementia with Lewy bodies (DLB) and Parkinson's disease dementia (PDD) have been presented in this table as an LBD group.
- Efforts have been made to account for overlapping cohorts, however in some instances this was not possible. Where there was uncertainty, groups have been reported separately and therefore there may be double-counting of included participants and total number of participants identified in this review may be over-estimated, particularly in the DLB and PDD groups. We made this decision (to over-estimate the sample size where there was uncertainty) to highlight the small sample sizes of LBD studies, including compared to other neurodegenerative diagnosis such as AD or PD.
- Clinical measures are listed only if the results are reported in the peer-reviewed publication. If a wider range of clinical assessments were performed in a study protocol but the results not provided, these are not listed.
- Country recorded in the first column refers to the geographical region from which participant samples were collected.

Table S1. Postmortem studies

Author, Year, Journal, Country	Title	Subjects, groups	Regions of interest at postmortem	Inflammation markers assessed & methods of assessment at postmortem	Outcomes/key results
McGeer, 1988, Neurology [1]<br><br>Canada	Reactive microglia are positive for HLA-DR in the substantia nigra of Parkinson's and Alzheimer's disease brains	LBD (5) / PD (5) / AD (9) / HC (7) / stroke (2)	Substantia nigra, hippocampus, temporal cortex, substantia innominate	IHC: HLA-DR for reactive microglia; GFAP for reactive astrocytes	Significant numbers of activated microglia were observed in the substantia nigra of all PD, LBD and, to a lesser extent, AD cases. Activated microglia were near melanin-containing dopaminergic cells and free melanin in tissue, and near degenerating dopaminergic cells in the substantia nigra.
Iseki, 2000, Neuroscience letters [2]<br><br>Japan	Degeneration process of Lewy bodies in the brains of patients with dementia with Lewy bodies using alpha-synuclein-immunohistochemistry	DLB (10)	Brainstem nuclei: pons (including locus coeruleus), medulla oblongata (including dorsal vagal nucleus)	IHC, immunoelectron microscopy: HDLA-DR, CD68 (phagocytic marker) and CR3/43 (HLA-DR/DP/DQ; antigen presentation) for activated microglia; GFAP for astrocytes; C1q, C3d and C4d for complement	Microglia were identified with degenerating Lewy body-containing neurons and late-stage engulfment of extracellular Lewy bodies, often found adjacent to blood vessels.<br>Astrocytes were associated with extracellular Lewy bodies. Occasional C3d and C4d immunoreactivity of intracellular and extracellular Lewy bodies pathology was observed, but not C1q, implicating the classical complement pathway.
Mackenzie, 2000, Neurology [3]<br><br>Canada	Activated microglia in dementia with Lewy bodies	'pure' DLB (5) / DLB with AD co-pathology (5) 'pure' AD (5) / HC (5)	Transentorhinal cortex, cingulum and frontal neocortex	IHC: HLA-DR/DP/DQ (MHC II) for activated microglia	More activated microglia were present in all dementia groups compared to controls.<br>There was a positive correlation between the number of activated microglial cells and the number of Lewy bodies in different brain regions, although there was no obvious tendency for activated microglia to aggregate around individual Lewy bodies or Lewy body-bearing neurons.
Rozemuller, 2000, Acta Neuropathologica 2000;100(6):701-8 [4]<br><br>The Netherlands	Activated microglial cells and complement factors are unrelated to cortical Lewy bodies	LBD (15) / PD (10) / AD (5) / young HC (5) / aged HC (4)	Cingulate gyrus	IHC: CD68, HLA-DR/DP/DQ for microglia with morphology assessment to determine activation state; C3c, C3d, C1q and C5-9 for complement	Microglia numbers in LBD tissue were no different from controls if there was no AD pathology also present, and activated microglia were not seen to associate with Lewy body-containing neurons. Complement factors were also not associated with α-synuclein pathology, whereas AD plaques did stain positively for all complement factors assessed.
Shepherd, 2000, Archives of Neurology [5]<br><br>Australia	Cortical inflammation in Alzheimer disease but not dementia with Lewy bodies	DLB (8) / AD (10) / HC (11)	Superior frontal, anterior cingulate, inferior temporal, hippocampus, para-hippocampal	IHC: HLA-DR for activated microglia	DLB brains, which contained no AD plaque co-pathology, had microglia distribution similar to controls and no well-defined clusters.

					In contrast, AD brains showed a significant burden of reactive microglia compared to controls, and these were aggregated in similar regions to the AD plaques.
Togo, 2001, Journal of the Neurological Sciences [6]<br><br>Japan	Glial involvement in the degeneration process of Lewy body-bearing neurons and the degradation process of Lewy bodies in brains of dementia with Lewy bodies	DLB (12)	Hippocampus, amygdala, temporal and cingulate cortices	IHC: HLA-DP/DQ/DR (CR3/43) for activated microglia; GFAP for astrocytes; TNF-α; complement (C4d); NF-kappaβ	Activated microglia frequently extended their processes to the cell membranes of degenerated neurons with Lewy bodies.<br>Some astroglial processes attached to extracellular Lewy bodies, and astroglia occasionally engulfed fragments of extracellular Lewy bodies.<br>C4d positivity associated with some intracellular Lewy bodies and AD co-pathology (amyloid deposits, extracellular neurofibrillary tangles).<br>Nuclear factor kappa B (NF-kB), a transcription factor activated by inflammatory cytokines, was implicated in intra-neuronal α-synuclein, whereas staining for TNF-α in microglia and astrocytes did not associate with neurons containing Lewy bodies.
Katsuse, 2003, Neuropathology [7]<br><br>Japan	Immunohistochemical study of the expression of cytokines and nitric oxide synthases in brains of patients with dementia with Lewy bodies	DLB (5) / AD (5) / HC (5)	Hippocampus (cornu ammonis (CA)1, CA2, CA3, CA4 and subiculum), amygdala, entorhinal, insular and middle temporal cortices, inferior parietal lobule	IHC: HLA-/DP/DQ/DR (CR3/43) for activated microglia; GFAP for astrocytes; IL-1α, TNF-α; inducible nitric oxide synthase (iNOS), neuronal NOS (nNOS)	iNOS, IL-1α and TNF-α expression was increased in the amygdala, hippocampus, entorhinal cortex and insular cortex of DLB brains, and nNOS was reduced in the amygdala of DLB brains.<br>Dual staining associated microglia and astrocytes with inflammatory molecule expression and disease pathology.<br>Intra-neuronal Lewy bodies were occasionally associated with the processes of IL-1α- and TNF-α-positive astroglia.<br>Most extracellular Lewy bodies were associated with TNF-α and iNOS-positive astroglia
Imamura, 2005, Acta Neuropathologica [8]<br><br>Japan	Cytokine production of activated microglia and decrease in neurotrophic factors of neurons in the hippocampus of Lewy body disease brains	DLB (5) / PD (5) / HC (4)	Cerebrum, brainstem, spinal cord, cerebellum; cell counts reported for hippocampus, amygdala, transentorhinal cortex	IHC: HLA-DP/DQ/DR (CR3/43) for activated microglia; GFAP for astrocytes.<br>mRNA expression via RT-PCR: IL-1α, IL-1β, TNF-α, IL-6, TGF-β	Microglia (CR3/43-positive) were diffuse in the hippocampus of PD and DLB brains and not control brains, and also significantly higher numbers were seen in all limbic system areas of PD/DLB compared to controls. No difference was observed in microglia between PD and DLB.<br>Only a few astrocytes were observed in PD brains.<br>IL-6 mRNA expression was significantly increased in the hippocampus, putamen and cingulate cortex of PD and DLB brains compared to controls; IL-1β was lower in PD and DLB hippocampus compared to controls, but higher in the cingulate cortex. TNF-α was higher in DLB cingulate cortex compared to controls.
Loeffler, 2006, Journal of	Complement activation in the Parkinson's disease	DLB (9) / AD (13) / PD (20) / young	Substantia nigra	IHC: iC3b (early-stage complement activation) and C9 (late-stage complement activation); measured	Complement staining of Lewy bodies was present in some, but not all, cases of PD (iC3b: 7 of 20, C9: 11 of 19) and DLB (iC3b: 6 of 9, C9: 9 of 9).

Neuroinflammation [9]<br><br>USA	substantia nigra: An immunocytochemical study	HC (13) / aged HC (28)		percentage of complement-positive melanised neurons	Complement staining in melanised neurons was not different in DLB compared to all other study groups.
Saldana, 2008, Movement Disorders [10]<br><br>Spain	Relevance of COX-2 gene expression in dementia with Lewy bodies associated with Alzheimer pathology	DLB (10) / HC (7)	Frontal cortex, substantia nigra pars compacta	RT-PCR & Western blot for COX-2 mRNA and protein expression, respectively	COX-2 protein levels were significantly lower in DLB compared to controls, and not detectable in the substantia nigra. No significant difference in COX-2 mRNA expression in either region was seen, although there was a trend towards decreased levels in the frontal cortex.<br>Nigral COX-2 mRNA or protein expression in DLB did not correlate with dopaminergic neurodegeneration.
Castellani, 2011, Journal of Neural Transmission [11]<br><br>USA	CD3 in Lewy pathology: does the abnormal recall of neurodevelopmental processes underlie Parkinson's disease	LBD (6) / PD (6) / HC (4)	Brainstem and neocortex	IHC: CD3ζ (CD3 subunit associated with T cells, NK cells, and dendritic cell outgrowth)	CD3ζ immunoreactivity was widespread in LBD and PD brains, not seen in control brains, and was associated with α-synuclein-containing Lewy body pathology. Therefore CD3 dysregulation may be an important mechanism in Lewy body disease pathogenesis.
Bachstetter, 2015, Acta neuropath communications [12]<br><br>USA	Disease-related microglia heterogeneity in the hippocampus of Alzheimer's disease, dementia with Lewy bodies, and hippocampal sclerosis of aging	DLB (12) / AD (7) / HC (9) / hippocampal sclerosis (HS; 7) / AD+HS (4)	Hippocampus	IHC: CD68 for phagocytic microglia, Iba1 for all microglia.<br>Quantitative image analysis of microglia morphology and number	Observed five morphologically-defined classes of Iba1 labelled microglia: ramified, hypertrophic, dystrophic, rod-shaped, and amoeboid.<br>Aged individuals without dementia were more likely to have ramified microglia than individuals with dementia.<br>In DLB, they observed low microglia density and increased proportion of dystrophic to all microglia.
Streit, 2016, Brain Behavior, and Immunity [13]<br><br>USA	Microglia in dementia with Lewy bodies	DLB (5) / HC (9)	Temporal pole, superior frontal gyrus	IHC: CD68 for phagocytic microglia, Iba1 for all microglia morphology assessment	Absence of activated microglia in all but two samples, based on detection of microglial hypertrophy and macrophage transformation.
Garcia-Esparcia, 2017, Frontiers in Neurology [14]<br><br>Spain	Dementia with Lewy Bodies: Molecular Pathology in the Frontal Cortex in Typical and Rapidly Progressive Forms	DLB (13; includes n=4 rapidly progressive DLB) / middle-aged HC (12)	Frontal cortex	IHC: CD68 and Iba1 for microglia; GFAP for astrocytes.<br>RT-qPCR for mRNA of several genes, including 23 genes encoding cytokines and inflammatory mediators (see Table 2)<br>Western blotting to quantify protein levels of TNF-α, Iba1 and GFAP from brain homogenate	Presence of mild-severe microgliosis and astrocytosis seen in DLB and rpDLB, which were absent in all controls.<br>No difference in neuropathology observed between DLB and rpDLB.<br>No difference found between controls and DLB of the 23 genes encoding cytokines and inflammatory mediators tested.<br>Protein quantification found increased GFAP for DLB and rpDLB compared to controls, significantly increased TNF-α in rpDLB compared to controls, with no differences in Iba1. mRNA levels of TNF-α was also higher in rpDLB compared to DLB.

Kohl, 2017, Neural Plasticity [15]<br><br>USA	Distinct pattern of microgliosis in the olfactory bulb of neurodegenerative proteinopathies	LBD (6) /<br>AD (10) /<br>FTLD (8) /<br>HC (6)	Olfactory bulb	IHC: CD68 for phagocytic microglia, Iba1 for all microglia morphology assessment<br><br>Clinical measures: MMSE (within 12 months prior to death)	Increased number of Iba1+ microglia observed in all dementia groups compared to controls, but no difference in number of activated microglia (determined by larger Iba1+ areas representing amoeboid cells).<br>No difference in CD68 expression in LBD compared to controls, whereas in AD it was increased.
*Llorens, 2017, Molecular Neurodegeneratio n [16]<br><br>Spain (brain tissue, CSF), Germany (CSF) and Greece (CSF)	YKL-40 in the brain and cerebrospinal fluid of neurodegenerative dementias	Postmortem:<br>DLB (20) /<br>AD (27) /<br>sCJD (45) /<br>HC (62)<br><br>*NB: postmortem cohort divided into two groups for separate analyses.*<br><br>CSF:<br>LBD (40) /<br>AD (84) /<br>VaD (20) /<br>sCJD (93) /<br>NC (62)	Frontal cortex	Postmortem:<br>IHC: IBA-1 for microglia; YKL-40, GFAP for astrocytes<br>RT-qPCR: YLK-40 expression<br><br>CSF:<br>EIA ELISA (commercial): YKL-40	Postmortem study:<br>YKL-40 and GFAP immunoreactivity associated with each other (most astrocytes showed both markers).<br>DLB:<br>• Trend towards difference in YKL-40 mRNA expression (n=9 DLB, n=4 rpDLB) and protein level (n=8) in DLB compared to controls (n=10 for qPCR, n=8 for IHC), but not statistically significant.<br>• Sparse YKL-40+ astrocyte clusters in cortical regions, white matter and subpial later, which were attributed to AD co-pathology.<br>AD:<br>• YKL-40 mRNA levels increased (compared to controls) in the frontal cortex, no difference at cerebellum.<br>• YKL-40 staining was mainly associated with astrocytes (GFAP+), which were clustered around fibrillar β-amyloid or blood vessels with β-amyloid.<br>*In vivo* CSF study:<br>YKL-40 levels in PDD/DLB (n=40) cases were not significantly different compared to neurological controls (n=62), although they were lower than sCJD cases.<br>YKL-40 was significantly higher in CSF of CJD compared to other dementias, and higher in CJD (n=104) and AD (n=84) compared to neurologic controls (n=62).
Chong, 2017, Journal of Alzheimer's Disease [17]<br><br>Norway and UK	Increased Transforming Growth Factor Beta2 in the Neocortex of Alzheimer's Disease and Dementia with Lewy Bodies is Correlated with Disease Severity and Soluble ABeta(42) Load	DLB (20) /<br>PDD (20) /<br>AD (14) /<br>HC	Temporal cortex - BA21	Luminex immunoassay (commercial):<br>TGFβ2<br><br>Clinical measures: yearly MMSE (subset of participants)	Increased TGFβ2 correlated with clinical and neuropathological markers of disease severity in DLB and AD:<br>• TGFβ2 concentrations were significantly elevated in DLB and AD compared to aged controls.<br>• Trend of elevation in PDD compared to controls but not significant.<br>• TGFβ2 levels correlated with MMSE decline, neurofibrillary tables and Lewy body pathology scores.

Wilhelmsson, 2017, Cerebral Cortex [18]<br><br>Sweden	Injury Leads to the Appearance of Cells with Characteristics of Both Microglia and Astrocytes in Mouse and Human Brain	LBD (10) / AD (10) / HC (10) / ischaemic stroke (14)	Temporal lobe	IHC microarray, immunofluorescence: Cx3cr1, AIF1 (aka Iba1), Itgam, and Cd68 for microglia; GFAP, S100β for astrocytes	Human study: co-expression of microglial (AIF1, aka Iba1) and astrocyte (S100β) markers by the same cell detected in a few cases across diagnostic groups (n=1 DLB, n=1 stroke, n=1 AD).
Walker, 2017, Neurobiology of Aging [19]<br><br>USA	Changes in CD200 and intercellular adhesion molecule-1 (ICAM-1) levels in brains of Lewy body disorder cases are associated with amounts of Alzheimer's pathology not alpha-synuclein pathology	DLB / PDD / PD / AD with LBs / iLB / HC<br><br>*NB: Group numbers not provided.*	Cingulate and temporal cortex	Sandwich ELISA, Western blot, IHC, RT-PCR: HLA-DR for microglial activation; CD200 anti-inflammatory marker; ICAM-1 used as a pro-inflammatory marker	Lewy body pathology did not correlate with CD200 or ICAM-1, and there was limited correlation between Lewy bodies and microglia. There was marked co-localisation between activated microglia and AD pathology (p-tau and amyloid- β).<br>Some differences in inflammatory markers were detected in DLB and PDD groups:<br>• Temporal cortex: DLB levels of ICAM1 were significantly higher than controls, whereas PDD was not different; no difference for CD200 between DLB/PDD and controls.<br>• Cingulate cortex: DLB CD200 significantly lower, and ICAM significantly higher, than controls; no difference for PDD or PD.<br>Clinical AD with LB pathology:<br>• Temporal cortex: significantly lower CD200 than controls, and significantly higher ICAM1 than controls.<br>• Cingulate cortex: lower CD200 and higher ICAM1<br>No differences found between AD-LB and DLB groups.
Ren, 2018, Proceedings of the National Academy of Sciences [20]<br><br>Japan	Soluble epoxide hydrolase plays a key role in the pathogenesis of Parkinson's disease	DLB (10) / HC (10)	Striatum	Western blot analysis of protein levels: soluble epoxide hydrolase (sEH)	Levels of sEH protein were significantly higher in the DLB cases compared to controls, and also correlated with the ratio of phosphorylated to unphosphorylated α-synuclein in brain tissue.
Erskine, 2018, Movement Disorders [21]<br><br>UK	Molecular changes in the absence of severe pathology in the pulvinar in dementia with Lewy bodies	DLB (25) / HC (27)<br><br>*NB: total cohort divided into two groups for separate analyses.*	Pulvinar	Transcriptomics cohort:<br>RNA sequencing for transcriptomics (mRNA), Western blot for protein expression; included CHI3L1 (aka YKL-40), GFAP, heat shock protein 70 1B (HSPA1B), SERPINH1/HSP47<br><br>Histopathology cohort:<br>IHC: HLA-DP/DQ/DR (M1 microglia), CD74 (cytotoxic M1 microglia), Iba1 for microglia; GFAP,	Transcriptomics cohort:<br>• The DLB group had higher protein and mRNA levels of CHI3L1 (aka YKL-40), GFAP, heat shock protein 70 1B (HSPA1B).<br>• mRNA levels of SERPINH1/HSP47 and heat shock protein 70 1A (HSPA1A) were also different between the groups, but there was no difference in protein expression.<br>Histopathology cohort:<br>• Lewy body pathological burden in the pulvinar was absent or mild, but many immunoreactive dots and fine threads were observed.

				aldehyde dehydrogenase family member 1 (ALDH1L1) for astrocytes<br><br>Clinical measures: 9/14 DLB cases underwent neuropsychological evaluation, including hallucination score	• Microglia expression or morphology was not different between the groups.<br>• Astrocyte markers were altered in DLB cases compared to controls, with increased GFAP that did not correlate with hallmark neuropathology (α-synuclein, amyloid-β or tau) or neuronal number, but did negatively correlate with synaptic markers.<br>Correlation with clinical hallucination score was only observed with tau burden, and not GFAP or any other neuropathological markers.
Santpere, 2018, Brain Pathology [22]<br><br>Spain	Transcriptional network analysis in frontal cortex in Lewy body diseases with focus on dementia with Lewy bodies	DLB (17) / PD (17) / HC (23) / iLBD (12)<br><br>*NB: includes study cohort and validation cohort.*	Frontal cortex area 8	Gene microarray analysis (gene enrichment scores, differentially expressed genes (DEGs), weighted gene co-expression network analysis (WGCNA))<br>RT-PCR for protein expression (of 50+ selected genes) to validate identified hubs and selected altered pathways	DEGs were identified in DLB compared to all other groups, with the most common finding being downregulation of genes in the DLB group compared to controls.<br>In DLB, deregulated cluster relating to inflammation and immune system functions included downregulation of antigen presentation and processing (including via MHC II) and the innate immune response activating cell surface receptor signalling pathway.<br>Weighted gene co-expression network analysis also identified DLB-associated modules that were enriched in neuronal and microglial markers.<br>RT-qPCR protein expression identified reduced AIF1 (encodes microglial marker Iba1) in iLBD compared to controls
Xu, 2019, Annals of Clinical and Translational Neurology [23]<br><br>USA	Translocator protein in late stage Alzheimer's disease and Dementia with Lewy bodies brains	DLB (5) / AD (7) / HC (8)	Right hemisphere: thalamus, substantia nigra, frontal cortex, caudate, putamen, red nucleus	Quantitative autoradiography: TSPO ligands [3H]PK11195 and [3H]PBR28 used to detect microglia<br><br>Clinical measures: CDR	Reduced TSPO density found in the substantia nigra of DLB and AD brains compared to age-matched healthy controls, which may be due to microglia dystrophy with reduced TSPO expression or lower microglial numbers in advanced disease.
de Wit, 2019, Journal of Neuroinflammatio n [24]<br><br>Netherlands	Astrocytic ceramide as possible indicator of neuroinflammation	PDD (5) / FTD-Pi (5) / HC (5)	Inferior frontal gyrus	IHC: HLA-DR for microglia; GFAP for astrocytes<br>Lipid extraction: sphingolipids (ceramide, acid sphingomyelinase, ceramide synthase 2 and 5, sphingosine, and sphingosine 1-phosphate)	No alterations in sphingolipid metabolism or immunoreactive area for microglia the PDD group were detected, although there were a few trends. Therefore the authors conclude that small sample sizes may have limited conclusions.
Amin, 2020, Translational Psychiatry [25]	Neuroinflammation in dementia with Lewy bodies: a	DLB (30) / HC (29)	Middle temporal gyrus (BA 21)	IHC: HLA-DR (antigen presentation), Iba1 (motility), CD68 (lysosomal phagocytosis) for microglia; CD3 for lymphocytes;	While DLB brain tissue contained marked neuropathology compared to controls, there was no difference in microglial (Iba1, HLA-DR, CD68), inflammatory (IL4R, CHI3L1 aka YKL-40), and several immune markers (CD64, CD32b).

UK	human post-mortem study			IL4R and CHI3L1 (YLK-40), as markers of alternative microglial activation; CD64, CD32a, CD32b and CD16 antibody receptors involved in immunoglobulin-mediated immune responses	CD32a load was significantly lower, and CD16 load higher, in DLB compared with controls.<br>Recruitment of CD3+ T lymphocytes into brain parenchyma was increased in DLB compared to controls.<br>The authors conclude these findings suggest altered homeostasis inflammatory mechanisms in DLB.
Iba, 2020, Journal of Neuroinflammation [26]<br><br>USA	Neuroinflammation is associated with infiltration of T cells in Lewy body disease and alpha-synuclein transgenic models	DLB (8) / HC (8)	Neocortex and hippocampus	IHC: CD3, CD4, CD8 for T lymphocytes; CD20 for B lymphocytes	Compared to controls, DLB brains were found to have increased numbers of infiltrating CD3+/CD4+ T cells in close proximity with blood vessels and neuropils.<br>A few CD8+ T cells were visualised in DLB hippocampus tissue, but none in neocortex or in tissue from controls.<br>CD20+ B cells were scarce in both groups.
Kim, 2020, Science Translational Medicine [27]<br><br>USA	LRRK2 mediates microglial neurotoxicity via NFATc2 in rodent models of synucleinopathies	DLB (10) / HC (8)	Neocortex and striatum	IHC, biochemical analysis of whole-brain lysates: IBA-1 for microglia; GFAP for astroglia; NFATc2	DLB brains had pronounced microgliosis and astrogliosis, and higher protein and gene expression of NFATc2, compared to controls.<br>NFATc2 was detected in cells that expressed neuronal (NeuN), astroglial (GFAP), or microglial (IBA-1) markers.
Kouli, 2020, Acta Neuropathologica Communications [28]<br><br>UK	Neuroinflammation and protein pathology in Parkinson's disease dementia	PDD (11) / PD (17) /HC (14)	Substantia nigra, amygdala, hippocampus, entorhinal, occipito-temporal, prefrontal, and posterior parietal cortex	IHC, digital quantification: HLA-DR, Iba1 and morphology assessment for microglial activation; GFAP for astrogliosis; CD4+, CD8+ for T lymphocytes<br>qRT-PCR (RNA): TNF-α, IL-1β, IL-6, IL-8, TLR2, TLR4.<br><br>Clinical measures: MMSE, UPDRS, Hoehn and Yahr	Some cell populations with inflammatory and immune functions were altered in some regions of PDD brains:<br>• Number of activated microglia was increased in PDD amygdala compared to controls and correlated with α-synuclein pathology. In hippocampal tissue from PDD cases, microglia were also increased compared to PDD but did not withstand multiple comparisons correction, and there was no difference in the substantia nigra between all groups.<br>• Astrogliosis (GFAP+) was increased in PD cases compared to controls, but there was no differences detected in the PDD group.<br>• CD4+ T lymphocytes were increased in amygdala and substantia nigra tissue from PDD and PD brains compared to controls, and PDD counts were greater than PD. In the amygdala, these findings correlated with activated microglia, α-synuclein and tau pathology.<br>• CD8+ lymphocytes were present most frequently in PDD cases, but this was not statistically significant across groups after correcting for multiple comparisons.<br>Gene expression of inflammatory cytokines and TLRs:

					• IL-1β levels were increased in PDD and PD cases in the substantia nigra and frontal cortex compared to controls, along with increased TLR4.<br>No differences detected for TNF-α, IL-6, IL-8 and TLR2 levels.
*Li, 2020, International Journal of Molecular Sciences [29] / Li, 2022, Neural Regeneration Research [30]<br><br>USA	Microglia implicated in tauopathy in the striatum of neurodegenerative disease patients from genotype to phenotype / Striatal oxidative damages and neuroinflammation correlate with progression and survival of Lewy body and Alzheimer diseases	DLB (10) / PDD (8) / PD (8) /AD (27) / HC (10)	Caudate and putamen	ELISA, autoradiography: TSPO for microglia; TREM2, MPO, PAR for microglia-related inflammation<br><br>Genotyping (SNPs): microglia-associated genes *BIN1*, *TREM2*, *TSPO*<br><br>Clinical measures: Hoehn and Yahr stage	Significant associations were identified between biochemical markers of inflammation, SNPs, microglia, tau neuropathology, and clinical measures of disease severity and progression in Lewy body dementia brains:<br>• Reduced microglia (TSPO) density and tau fibrils were observed in the Lewy body disease (DLB/PDD/PD) cohort compared with controls, and positive correlations existed between the concentration of tau fibrils and MPO.<br>• TSPO level in tissue was also haplotype dependent, with certain polymorphisms in TSPO appearing to confer increased or reduced levels.<br>• TREM2 levels increased with disease progression in the putamen of Lewy body disease brains, and there was a significant negative association between TREM2 expression and tau fibrils in DLB cases. TREM2 expression in the caudate was influenced by allele G of the *BIN1* gene, therefore this allele may be a risk factor for tauopathy.<br>• PAR concentration in the caudate of Lewy body disease brains, including PDD and DLB when analysed separately, was positively associated with disease progression.
Rajkumar, 2020, American Journal of Geriatric Psychiatry [31]<br><br>UK	Postmortem Cortical Transcriptomics of Lewy Body Dementia Reveal Mitochondrial Dysfunction and Lack of Neuroinflammation	DLB (7) / PDD (7) / HC (7)	Anterior cingulate, dorsolateral prefrontal cortices	Next-generation RNA-sequencing for transcriptomics of brain tissue. High-throughput qPCR to verify identified DEGs	In the combined LBD (DLB/PDD) cohort, compared to controls, down-regulation of several inflammatory genes was verified by qPCR. These included IL-1β, CXCL11 (a chemokine), neutrophil defensin genes (DEFA3, DEFA4) and VCAM-1. Twelve novel DEGs (MPO, SELE, CTSG, ALPI, ABCA13, GALNT6, SST, RBM3, CSF3, SLC4A1, OXTR, and RAB44) were identified and verified in LBD brains with genome-wide statistical significance.<br>Analysis for dysfunctional molecular networks implicated immunosenescence in LBD pathology, by finding significant enrichment in genes associated with granulocytes adhesion and diapedesis, regulation of cytokine production in macrophages and T-helper cells by IL-17A and IL-17F pathways, IL-6 signalling pathways, and communication between innate and adaptive immune cells.
Gate, 2021 Science [32]	CD4+ T cells contribute to neurodegeneration	Total study: DLB (12) / PDD (46) /	Substantia nigra, meninges	Postmortem:<br>IHC: CD3 for T cells, CD4 for helper T cells; Iba1 for innate immune cells;	Postmortem:<br>• LBD brain tissue had higher numbers of CD3+ T cells compared to controls, and these T cells were bound to Iba1+

USA	in Lewy body dementia.	PD (92) / HC (162)<br><br>Postmortem study: LBD (7) / HC (5)<br><br>*In vivo* CSF study: PDD (32) / PD (36) / HC (52)		IL-17A (inflammatory cytokine); CXCR4, CXCR12 (chemokines involved in T cell signalling)<br><br>*In vivo* CSF:<br>CXCR12 (chemokine; ligand for CXCR4)<br><br>Single-cell RNA sequencing (scRNAseq) analyses also performed on blood and CSF samples from a PD-DLB subset (n=11), although DLB results are not reported separately, therefore data is not extracted<br><br>Clinical measures: MoCA, UPDRS-motor, Hoehn and Yahr, Levodopa equivalency daily dose	innate immune cells and associated with α-synuclein deposits and Lewy bodies.<br>• CD4+IL-17A+ T cells were observed in PDD brains adjacent to IL-17A+ dopaminergic neurons, and IL-17A immunoreactivity levels were higher in LBD brains compared to controls.<br>• CD3+ T cells were adjacent to CXCL12+ vessels in the perivascular space, and CD3+CXCR4+ cells were also co-localised with CXCL12 in the meninges.<br>*In vivo* CSF:<br>• CXCR12 levels in the PDD group correlated with the neurodegeneration biomarker neurofilament light, although there was no significant difference in the levels between the groups.<br>With the addition of additional analyses involving other human study subsets (e.g., PD-DLB scRNAseq) and a murine model of α-synucleinopathy, the authors propose that dysregulated CXCR4-CXCR12 signalling is involved with CD4+ T cell recruitment to brain tissue and neurodegeneration in LBD, of which IL-17A production is a pathogenic mechanism.
Low, 2021, Brain Pathology [33]<br><br>Norway and UK	Isoform-specific upregulation of FynT kinase expression is associated with tauopathy and glial activation in Alzheimer's disease and Lewy body dementias	DLB (39) / PDD (27) / AD (13) / HC (16)	Frontal (BA9, dorsolateral/ medial prefrontal cortex) and temporal (BA21: middle temporal gyrus) cortex	Microarray for high-throughput transcriptome profiling: FynT, FynB. RT-PCR for gene expression: CD11b for microglia, GFAP for astrocytes<br><br>Clinical measures: MMSE	Microglial marker CD11b was increased in BA21 for all dementia groups, including PDD and DLB, whereas astrogliosis (GFAP expression) was increased in BA21 for DLB and not altered in PDD in any of the regions assessed.<br>In the combined dementia cohort, FynT expression correlated with both GFAP and CD11b. Selective upregulation of FynT expression in DLB, PDD and AD groups correlated with worse dementia severity.
Terreros-Roncal, 2021, Science [34]<br><br>Spain	Impact of neurodegenerative diseases on human adult hippocampal neurogenesis	DLB (6) / PD (3) / FTD (6) / ALS (12) / HD (6) / HC (15)	Hippocampus	IHC: Iba1 for microglia (including visualisation of phagocytic pouches), S100β for astrocytes	Alterations in microglia were observed in all study groups, including a partially dysfunctional phenotype even in the control group, leading the authors to conclude that there is an age-related decline in microglial function. In the DLB and PD groups, decreased phagocytic capacity of microglia was identified, as well as astrogliosis in the PD group.
Chua, 2022, Neurochemistry International [35]	Elevation of inactive cleaved annexin A1 in the neocortex is associated with amyloid, inflammatory and	DLB (24) / PDD (17) / AD (13) / HC (16)	Frontal cortex (BA9), parietal lobe (BA40)	Immunoblotting (protein quantification): Annexin A1 (AxnA1; an anti-inflammatory signalling molecule).<br>RT-PCR (mRNA quantification): AnxA1	Increased levels of inactive cleaved AnxA1, but not the active un-cleaved form, were found in DLB and AD parietal lobe tissue, compared to controls. This was associated with increased soluble β-amyloid load but not with severity of Lewy body disease-associated α-synucleinopathy. No difference was seen in the PDD group.

	apoptotic markers in neurodegenerative dementias			Luminex assay (protein concentration): IL-1α, IL-13, IL-10<br><br>Clinical measures: MMSE (before death and yearly decline)	Increased levels of all measured pro- and anti-inflammatory cytokines (IL-1α, IL-13, IL-10), as well as apoptosis-associated molecule caspase-3, were correlated with the levels of cleaved AnxA1 in the parietal tissue. The authors therefore conclude that inactivation of the anti-inflammatory molecule AxnA1 results in failure of regulatory homeostatic processes in amyloid-associated disease and may contribute to neurodegeneration. This potential pathogenic mechanism was identified in DLB but not PDD brains. Absolute levels of cytokines in each group were not reported.
Fixemer, 2022, Acta Neuropathologica Communications [36]<br><br>Canada and Luxembourg	Microglia phenotype s are associated with subregional patterns of concomitant tau, amyloid-beta and alpha-synuclein pathologies in the hippocampus of patients with Alzheimer's disease and dementia with Lewy bodies	DLB (8) / AD (10) / HC (11)	Hippocampus (CA1, CA3 and DG/CA4 subfields)	IHC: Iba1 for microglial morphology, which was determined using an automated pipeline that classified microglia based on 16 morphological features, including sorting into 7 distinct morphological clusters	Similar alterations in microglial morphology overall and clusters were detected in both DLB and AD groups compared to controls, including increased 'compactness' suggesting more amoeboid morphologies, however these differences were only statistically significant in the AD group.<br>Close associations were also seen in both DLB and AD groups between microglial morphology alterations and hippocampal subfield volume of association neuropathology (phosphorylated tau, amyloid-β and phosphorylated α-synuclein), which was generally greater in the AD group.
Tu, 2022, BMC Neuroscience [37]<br><br>UK	Increased expression of pathological markers in Parkinson's disease dementia post-mortem brains compared to dementia with Lewy bodies	DLB (9) / PDD (10) / HC (9)	Substantia nigra, temporal cortex, caudate and putamen	Western blot (protein quantification): Iba1 for microglial-associated neuroinflammation, GFAP for astrocyte activity	In the temporal cortex, Iba1 levels were significantly increased in the PDD group compared to controls, and GFAP levels were significantly elevated in the DLB group.<br>No other differences in Iba1 or GFAP protein levels between the study groups were reported.

Table S2. Imaging

**Author, Year, Journal**	**Title**	**Subjects, groups**	**Regions of interest (ROI) on imaging**	**Technique to assess inflammation**	**Outcomes/key results**
Iannaccone, 2013, Parkinsonism & Related Disorders [38]<br><br>Spain	In vivo microglia activation in very early dementia with Lewy bodies, comparison with Parkinson's disease	DLB (6) / PD (6) / HC (11)	Frontal lateral cortex, parietal lateral cortex, temporal lateral cortex, temporal pole, precuneus, occipital medial and lateral cortices, anterior and posterior cingulate cortices, cerebellum, hippocampus, amygdala, caudate, putamen, thalamus, substantia nigra	PET: [11C]-PK11195 for TSPO (microglia)<br><br>Clinical measures: MMSE, UPDRS-III, Hoehn and Yahr	PET detected microglia-related inflammation in DLB and PD at the substantia nigra and putamen, early in the disease course.<br>The authors acknowledge that this pilot study was limited by small sample size.
Fan, 2015, Alzheimer's & Dementia [39] /<br>Edison, 2013, Neuropsychopharmacology [40]<br><br>UK	Influence of microglial activation on neuronal function in Alzheimer's and Parkinson's disease dementia / Microglia, amyloid, and glucose metabolism in Parkinson's disease with and without dementia	PDD (11) / AD (10) / MCI (10) / HC (16)	48 ROIs across left and right hemispheres; see Table 1A in Fan et al. 2015 for full list.	PET: [11C](R)PK11195 for TSPO (microglia)<br><br>Clinical measures: MMSE	In PDD, compared to controls, [11C]-PK11195 PET microglial activation is associated with reduced glucose metabolism on PET, and with reduced cognitive scores in both cohorts.
Femminella, 2016, Journal of Alzheimer's Disease [41]<br><br>UK	Does Microglial Activation Influence Hippocampal Volume and Neuronal Function in Alzheimer's Disease and Parkinson's Disease Dementia?	PDD (9) / AD (8) / HC (8)	40+ ROIs including right and left hemispheres; see Tables 2 and 3 for full list.	PET: [11C](R)PK11195 for TSPO (microglia)<br><br>Clinical measures: MMSE and several other cognitive assessment tools	[11C]-PK11195 PET binding was associated with reduced hippocampal volumes in PDD and AD compared to controls, and negatively correlated with cognitive scores in the whole study cohort.
*Surendranathan, 2018, Brain [42] / Nicastro 2020, NeuroImage - Clinical [43]<br><br>NIMROD study<br><br>UK	Early microglial activation and peripheral inflammation in dementia with Lewy bodies / Correlation of microglial activation with white matter changes in dementia with Lewy bodies	DLB (19) / HC (26)	40+ ROIs including right and left hemispheres; see Figure 1 in Surendranathan et al. for full list.	PET: [11C]-PK11195 for TSPO (microglia)<br><br>Blood: Electrochemiluminescence immunoassay, ELISA (multiplex assay): included hsCRP, IL-2, IL-6, TNF-α, IL-12, IL-15, IFNγ, IL8, MCP-1, MIP-	PET:<br>• [11C]-PK11195 binding was elevated in DLB subjects with mild disease (based on cognitive scores) compared to those with moderate/severe disease. Relative preservation of brain white matter on MRI associated with increased microglial activation, indicating that inflammation is an early event in DLB.

**Author, Year, Journal**	**Title**	**Subjects, groups**	**Regions of interest (ROI) on imaging**	**Technique to assess inflammation**	**Outcomes/key results**
				3a, IP10, VEGF, TNF-R1, IL-34, YKL-40 (see Table 2 in *Surendranathan et al*. for list)<br><br>Clinical measures: ACE-R, MMSE, UPDRS-III	Blood inflammatory molecules:<br>• T lymphocyte-associated cytokines MIP-3a and IL-17a levels were increased, and IL-8 levels were decreased, in DLB participants compared to controls.
Nicastro, 2019, Annals of Clinical and Translational Neurology [44]<br><br>NIMROD study<br><br>UK	11 C-PK11195 PET imaging and white matter changes in Parkinson's disease dementia	PDD (5) /*HC (16)<br><br>*Control data from the NIMROD study reported by *Surendranathan et al*. used as the comparator.	ROIs not specifically stated; references NIMROD study protocol reported above by *Surendranathan et al*.	PET: [11C]-PK11195 for TSPO (microglia)<br><br>Clinical measures: ACE-R, MMSE, UPDRS-III	[11C]-PK11195 binding in PDD subjects was similar or lower than controls when analysed by region or as the whole cortex.<br>Higher [11C]-PK11195 binding in PDD cases was associated with a relative preservation of white matter integrity, suggesting microglia-related inflammation is important early in the PDD disease course.<br>No association was found between motor parkinsonism severity (UPDRS-III score) and [11C]-PK11195 binding.

Table S3. Cerebrospinal fluid

Author, Year, Journal	Title	Subjects, groups	Inflammation markers assessed & methods of assessment in CSF	Outcomes/key results
Gomez-Tortosa, 2003, Archives of Neurology [45]<br><br>Spain	Cerebrospinal fluid markers in dementia with Lewy bodies compared with Alzheimer disease	DLB (25) / AD (33) / HC (46)	ELISA (commercial kits): IL-1β, IL-6<br><br>Clinical measures: MMSE	No difference in IL-1β or IL-6 levels detected between DLB and AD, or compared to age-matched controls.
Janssen, 2004, Journal of Neurology [46]<br><br>UK	The prevalence of oligoclonal bands in the CSF of patients with primary neurodegenerative dementia	DLB (7) / AD (70) / FTLD (47) /other dementia (7)	Agarose isoelectric focusing: oligoclonal bands (OCB)	Nine out of 131 dementia cases had CNS-specific OCB detected, but none of the DLB cases did.
*Rota, 2006, Neurological Sciences [47]<br><br>Italy	Increased intrathecal TGF-beta1, but not IL-12, IFN-γ and IL-10 levels in Alzheimer's disease patients	PDD (10) /PD (14) / AD (30) / VaD (19) / NPH (14) / HC (25)	ELISA RIAs (in-house): IL12 p40, IL-12 p70, IFN-γ, TGF-β1<br>ELISA (commercial): IL-10, TGF-β1<br><br>Clinical measures: MMSE.	No significant differences were found in the PDD group for any of the assessed markers. TGF-β1 was increased in CSF of AD cases but not serum.<br>No correlation was found between the paired measurements for each cytokine in CSF and serum.
*Ernst, 2007, Journal of Neuroimmunology [48]<br><br>Germany	Procalcitonin is elevated in the cerebrospinal fluid of patients with dementia and acute neuroinflammation	DLB (8) / AD (40) /VascD (12) / FTD (12) / HC (50) / acute neuro-inflammation (16)	Highly sensitive commercial assay for procalcitonin [49] (same for CSF and blood)	Procalcitonin levels in CSF were significantly elevated in DLB (and all other dementia groups) compared to controls, whereas plasma levels were not different to controls. CSF and plasma levels of procalcitonin did not correlate in the dementia groups, but did in the acute inflammation group. Therefore, increase in CSF:blood ratio of procalcitonin in dementia may reflect subacute or chronic inflammation in the brain.
*Maetzler, 2007, Neurobiology of Disease [50]<br><br>Germany	Osteopontin is elevated in Parkinson's disease and its absence leads to reduced neurodegeneration in the MPTP model	PDD (9) / PD (21) / HC (30)	ELISA (commercial): osteopontin (same for CSF and blood)<br><br>Clinical measures: Hoehn and Yahr	Osteopontin levels in serum and blood were significantly higher in PD/PDD compared to controls, and participants with PDD has significantly higher osteopontin in CSF, but not blood, compared to PD.
*Nielsen, 2007, Neurobiology of Disease [51] / Nielsen, 2007, Neurology [52] / Nielsen, 2012, Current Alzheimer Research [53] /	Soluble adhesion molecules and angiotensin-converting enzyme in dementia / Plasma and CSF serpins in Alzheimer disease and dementia with Lewy bodies / Gender-dependent levels of hyaluronic acid in cerebrospinal fluid of	DLB (39) / AD (260) / HC (34)	ELISA (commercial): sICAM-1, sVCAM-1, sPECAM-1, ACE, hyaluronic acid (HA), MCP-1 (CSF and blood)<br>ELISA (in-house): neuroserpin (CSF only)<br><br>Rocket immunoelectrophoresis (in-	Vascular inflammatory markers:<br>• Compared to controls, both dementia groups had significantly altered endothelial marker levels in plasma (increased sICAM-1, increased sPECAM-1) and CSF (lower sVCAM1). The DLB group was distinguished from the AD group by higher sICAM-1 in plasma and CSF, and higher plasma sPECAM-1.<br>Serpins (AAT, ACT, neuroserpin; implicated in inflammation modulation):

Author, Year, Journal	Title	Subjects, groups	Inflammation markers assessed & methods of assessment in CSF	Outcomes/key results
Wennstrom, 2014, International Journal of Tryptophan Research [54]<br><br>Malmo Alzheimer Study<br><br>Sweden	patients with neurodegenerative dementia / Kynurenic Acid levels in cerebrospinal fluid from patients with Alzheimer's disease or dementia with Lewy bodies		house modifications): AAT, ACT (CSF and blood)<br><br>Liquid chromatography: kynurenic acid (CSF only)<br><br>Clinical measures: MMSE	• In the DLB group, worse cognition (lower MMSE scores) was associated with higher levels of AAT and t-tau in CSF.<br>• CSF serpin levels were elevated in DLB and AD groups compared to controls, and in plasma for the AD group. In models using these serpins for discrimination of DLB from AD, diagnostic performance was not better than measuring the standard diagnostic markers t-tau, p-tau and Aβ1-42<br>Hyaluroinic acid:<br>• No significant difference in levels between controls and DLB or AD groups.<br>• CSF levels of hyaluroinic acid were different between males and females in both dementia groups, and in females correlated positively with other inflammatory markers.<br>MCP-1 and kynurenic acid:<br>• CSF MCP-1 levels were not different between the DLB, AD and control groups. There was no effect of age or gender on levels, and MCP-1 did not correlate with kynurenic acid levels in CSF.
*Maetzler, 2009, Journal of Neural Transmission [55]<br><br>Germany	A single-nucleotide polymorphism of the osteopontin gene may contribute to a susceptibility to Lewy body disease	DLB (9) / PDD (6) / PD (43) / HC (30)	ELISA (commercial): osteopontin (CSF and blood)<br>Commercial Primer-Extension Assay for genotyping: osteopontin allele polymorphisms SNP-66 and SNP 1239.<br><br>Clinical measures: Hoehn and Yahr	Osteopontin levels were increased in CSF and blood of Lewy body disease (DLB/PDD/PD) participants compared to controls. After adjusting for age, there was no difference in osteopontin levels between DLB, PDD and PD in CSF and blood.<br>Discrimination ability of osteopontin between Lewy body disease and controls was moderate for CSF (AUC = 0.74) and poor (AUC = 0.68) for serum.<br>The SNP-66 osteopontin gene polymorphism was associated with Lewy body disease, but the genotype prevalence did not significantly correlate with osteopontin levels in CSF or blood.
Jesse, 2011, Journal of Neurology [56]<br><br>Germany	Summary of cerebrospinal fluid routine parameters in neurodegenerative diseases	PDD (25) / PD (47) / AD (289) / VaD (13) / HC (83) / MSA (25) / PSP (16) / CBD (5) / HD (31) / FTLD (28) / SCA (6) / MND (177) / MD (20)	Isoelectric focusing on agarose gels and subsequent immunoblotting (commercial): IgG oligocolonal bands, IgG, IgA, IgM	No alterations in oligoclonal bands, routine CSF parameters (leukocytes, albumin, lactate) or CSF-blood barrier dysfunction were found in the range of neurodegenerative diseases studied, including PDD.
Wang, 2011, The American Journal of Pathology [57]	Complement 3 and factor H in human cerebrospinal fluid in Parkinson's disease,	PDD (11) / PD (49) / PD-CIND (65) /AD (50) /	Luminex assay (commercial): C3 (complement 3), FH (factor H)	There was no significant difference in CSF levels of C3 or FH between the combined PD group (PDD/PD/PD-CIND) and controls.

**Author, Year, Journal**	**Title**	**Subjects, groups**	**Inflammation markers assessed & methods of assessment in CSF**	**Outcomes/key results**
USA	Alzheimer's disease, and multiple-system atrophy	MSA (32) /HC (137)	Clinical measures: MMSE (AD group only), UPDRS (PD only)	In PDD, ratio of C3/Aβ42 and FH/Aβ42 was significantly increased compared to PD participants without dementia (some had cognitive impairment). However, Aβ42 was significantly decreased in PDD compared to PD and PD-CIND groups, therefore AD co-pathology may be impacting this result.<br>The MSA group was distinguished from the combined PD cases and controls by increased levels of C3 and increased C3/FH ratio.
Wennstrom, 2015, PLOS ONE [58] / Wennstrom, 2015, Alzheimer's Research & Therapy [59]<br><br>Sweden	The inflammatory marker YKL-40 is elevated in cerebrospinal fluid from patients with Alzheimer's but not Parkinson's disease or dementia with Lewy bodies / Cerebrospinal fluid levels of IL-6 are decreased and correlate with cognitive status in DLB patients	DLB (36) /PD (61) / AD (49) /HC (44)	ELISA (commercial): YKL-40, GFAP. Electrochemiluminescence multiplex immunoassay (commercial): IL-6, TNF-α, IFN-γ, IL-1β<br><br>*NB: the PD group was not included in the analysis of IL-6, TNF-α, IFN-γ, and IL-1β*<br><br>Clinical measures: MMSE	Levels of astrocytic marker YKL-40 in CSF were significantly lower in DLB, PD and controls compared AD, and was not different between DLB and PD or controls.<br>YKL-40 levels did not correlate with GFAP levels.<br><br>IL-6 CSF levels in DLB cases were lower compared to controls, negatively correlated with cognition scores and positively correlated with CSF α-synuclein. Other inflammatory cytokines (TNF-α, IFN-γ, IL-1β) in the multiplex assay used were not reported as they were unable to be detected reliably across study groups.
Janelidze, 2016, Annals of Clinical and Translational Neurology [60]<br><br>Sweden	Cerebrospinal fluid neurogranin and YKL-40 as biomarkers of Alzheimer's disease	LBD (47) / AD (74) / HC (53) / stable MCI (62) / AD-MCI (35) / VaD (34) / FTD (33)	ELISA (commercial): YLK-40<br>Sandwich ELISA (in-house): neurogranin<br><br>Clinical measures: MMSE	YKL-40 and neurogranin levels in CSF of the LBD group were no different from controls, but YKL-40 levels were significantly lower compared to AD and FTD groups, and neurogranin levels were significantly lower than AD and AD-MCI groups.
*Llorens, 2017, Molecular Neurodegeneration [16]<br><br>Spain (brain tissue, CSF), Germany (CSF) and Greece (CSF)	YKL-40 in the brain and cerebrospinal fluid of neurodegenerative dementias	Postmortem: DLB (20) / AD (27) / sCJD (45) / HC (62)<br><br>*NB: postmortem cohort divided into two groups for separate analyses.*<br><br>CSF:<br>LBD (40) / AD (84) / VaD (20) / sCJD (93) / NC (62)	Postmortem:<br>IHC: IBA-1 for microglia; YKL-40, GFAP for astrocytes<br>RT-qPCR: YLK-40 expression<br><br>CSF:<br>EIA ELISA: YKL-40 (commercial)	Postmortem study:<br>YKL-40 and GFAP immunoreactivity associated with each other (most astrocytes showed both markers).<br>DLB:<br>• Trend towards difference in YKL-40 mRNA expression (n=9 DLB, n=4 rpDLB) and protein level (n=8) in DLB compared to controls (n=10 for qPCR, n=8 for IHC), but not statistically significant.<br>• Sparse YKL-40+ astrocyte clusters in cortical regions, white matter and subpial later, which were attributed to AD co-pathology.<br>AD:<br>• YKL-40 mRNA levels increased (compared to controls) in the frontal cortex, no difference at cerebellum.<br>• YKL-40 staining was mainly associated with astrocytes (GFAP+), which were clustered around fibrillar β-amyloid or blood vessels with β-amyloid.<br>*In vivo* CSF study:

Author, Year, Journal	Title	Subjects, groups	Inflammation markers assessed & methods of assessment in CSF	Outcomes/key results
				YKL-40 levels in PDD/DLB (n=40) cases were not significantly different compared to neurological controls (n=62), although they were lower than sCJD cases.<br>YKL-40 significantly higher in CSF of CJD compared to other dementias, and higher in CJD (n=104) and AD (n=84) compared to neurologic controls (n=62).
Del Campo, 2018, Annals of Clinical and Translational Neurology [61]<br><br>USA and Italy	Novel CSF biomarkers to discriminate FTLD and its pathological subtypes.	DLB (29) / AD (30) / HC (29) / FTLD-TDP (30) / FTLD-Tau (20)	EIA ELISA: YKL-40 (commercial)	YKL-40 CSF levels in the DLB group were significantly lower than the FTLD-TDP group, and no different from any other groups tested, including no difference compared to AD or healthy controls.
Hall, 2018, Scientific Reports [62] / Janelidze, 2015, Neurology [63] / Lindqvist, 2013, Brain, Behaviour, and Immunity [64]<br><br>BioFINDER study *(see also below: Ayton, 2022)*<br><br>Sweden	Cerebrospinal fluid concentrations of inflammatory markers in Parkinson's disease and atypical parkinsonian disorders / Increased CSF biomarkers of angiogenesis in Parkinson disease / Cerebrospinal fluid inflammatory markers in Parkinson's disease - Associations with depression, fatigue, and cognitive impairment	PDD (27) / PD (131) / HC (50) / MSA (24) / PSP (14)	Electrochemiluminescence multi-plex immunoassay (commercial): IL-8, MCP-1, CRP, IL-6, TNF-α, IP-10, MIP-1β, MCP-1, serum amyloid A (SAA)<br><br>Clinical measures: MMSE, UPDRS-III, Hoehn and Yahr, HADS, and several other cognitive assessment tools	CSF levels of SAA were higher in PDD compared to PD and controls, and CRP levels were higher in PDD compared to PD. CRP and SAA correlated with worse motor and cognitive performance in the PD group.<br>MCP-1 concentrations were lower in the PDD group compared to PD. When the PD/PDD groups were combined, MCP-1 levels were positively associated with angiogenesis markers (vascular endothelial growth factor (VEGF), placental growth factor (PlGF)).<br>In a smaller sample of participants within this cohort, IL-8 levels were higher in the PDD (n=18) group compared to controls, but not different from PD.<br>No difference was found between PDD and the other groups for CSF levels of IL-6, TNF-α, IP-10 and MIP-1β .<br>Several correlations between the inflammatory markers and participant characteristics were identified, including: age (higher MCP-1, IL-10, CRP), sex (higher MCP-1 in men), somatic illness score (higher CRP), motor symptoms (higher CRP, MCP-1), PD illness duration (higher CRP) and H&Y stage (higher CRP, MIP-1β).<br>Cognitive severity (MMSE) correlated with increased IL-6 levels in the PD/PDD group, and there was an association between anxiety or depression severity and the inflammatory markers MCP-1, IP-10 and CRP.
Paterson, 2018, Alzheimer's Research & Therapy [65]<br><br>UK	Cerebrospinal fluid in the differential diagnosis of Alzheimer's disease: clinical utility of an extended panel of biomarkers in a specialist cognitive clinic	DLB (20) / AD (156) / bvFTD (45) / PNFA (17) / SD (7) / HC (30)	Immunoassay: YKL-40<br><br>Clinical measures: MMSE	YKL-40 levels in CSF were higher in DLB, and all other dementias studied, compared to controls. There was no significant difference between DLB and AD levels of YKL-40.

Author, Year, Journal	Title	Subjects, groups	Inflammation markers assessed & methods of assessment in CSF	Outcomes/key results
Hu, 2019, Frontiers in Immunology [66]<br><br>USA	CSF Cytokines in Aging, Multiple Sclerosis, and Dementia	DLB (23) / PD (37) / AD (52) / MS (18) / HC (105; 53 < 60 years, 52 ≥ 60 years)	Luminex assay (commercial): TNF-α, IL-10, IL-8, IP-10	DLB and PD groups had higher CSF levels of TNF-α and lower IL-8 compared to controls.<br>In healthy controls, TNF-α, IP-10 and IL-8 levels all increased with age while controlling for gender. There was evidence for an age-related phenotype switch from Th1 to innate immunity and Th2 phenotypes, based on a relatively greater increase in these cytokines (TNF-a, IL-10, and IL-8) for each standard unit of age-associated increase in IP-10.
Morenas-Rodriguez, 2019, Scientific Reports [67]<br><br>Spain	Different pattern of CSF glial markers between dementia with Lewy bodies and Alzheimer's disease	DLB (15) / AD (57) / HC (24) / prod-DLB (6) / aMCI (43)	ELISA: YKL-40, soluble TREM2 (sTREM2), progranulin<br><br>Clinical measures: MMSE	YKL-40, sTREM2 and progranulin levels did not differ between DLB groups and controls.<br>YKL-40 levels were lower in DLB and prodromal DLB groups compared to AD and prodromal AD.<br>Patients with DLB with a CSF profile suggestive of AD co-pathology had higher levels of YKL-40, but not sTREM2 or PGRN, than those without.<br>Only YKL-40 correlated with t-tau and p-tau in DLB and in prodromal DLB.<br>Therefore, AD co-pathology may be the driver of increased YKL-40 levels in DLB cases.
Gate, 2021<br>Science [32]<br><br>USA	CD4+ T cells contribute to neurodegeneration in Lewy body dementia.	DLB (12) / PDD (46) / PD (92) / HC (162)<br><br>Postmortem:<br>LBD (7) / HC (5)<br><br>*In vivo* CSF:<br>PDD (32) / PD (36) / HC (52)<br><br>*NB: subgroups of the overall cohort were used for specific inflammation-related postmortem and CSF analyses.*	Postmortem:<br>IHC: CD3 for T cells, CD4 for helper T cells; Iba1 for innate immune cells; IL-17A (inflammatory cytokine); CXCR4, CXCR12 (chemokines involved in T cell signalling)<br><br>*In vivo* CSF:<br>CXCR12 (chemokine; ligand for CXCR4)<br><br>Single-cell RNA sequencing (scRNAseq) analyses also performed on blood and CSF samples from a PD-DLB subset (n=11), although DLB results are not reported separately therefore data not extracted<br><br>Clinical measures: MoCA, UPDRS-motor, Hoehn & Yahr, Levodopa equivalency daily dose	Postmortem:<br>• LBD brain tissue had higher numbers of CD3+ T cells compared to controls, and these T cells were bound to Iba1+ innate immune cells and associated with α-synuclein deposits and Lewy bodies.<br>• CD4+IL-17A+ T cells were observed in PDD brains adjacent to IL-17A+ dopaminergic neurons, and IL-17A immunoreactivity levels were higher in LBD brains compared to controls.<br>• CD3+ T cells were adjacent to CXCL12+ vessels in the perivascular space, and CD3+CXCR4+ cells were also co-localised with CXCL12 in the meninges.<br>*In vivo* CSF:<br>• CXCR12 levels in the PDD group correlated with the neurodegeneration biomarker neurofilament light, although there was no significant difference in the levels between the groups.<br>With the addition of additional analyses involving other human study subsets (e.g., PD-DLB scRNAseq) and a murine model of α-synucleinopathy, the authors propose that dysregulated CXCR4-CXCR12 signalling is involved with CD4+ T cell recruitment to brain tissue and neurodegeneration in LBD, of which IL-17A production is a pathogenic mechanism.

Author, Year, Journal	Title	Subjects, groups	Inflammation markers assessed & methods of assessment in CSF	Outcomes/key results
Lourenco, 2021, Journal of Alzheimer's Disease, [68]<br><br>Brazil	Cerebrospinal fluid neurotransmitters, cytokines, and chemokines in Alzheimer's and Lewy body diseases	LBD (9) / aMCI (14) / AD (14) / HC (25)	Luminex immunoassay (commercial): IL-8, IP10, MCP-1, MIP-1a, RANTES, and VEGF<br>*NB: a further 24 analytes including chemokines and cytokines (TNF-α IFN-γ, IL-1β, IL-2, IL-4, IL-6, IL-10, IL-17 etc.) were analysed in the same multiplex immunoassay but were not reliably detected.*<br><br>Clinical measures: MMSE	Of the six inflammation-associated molecules that were reliably detected in CSF, the only difference detected between control and neurodegenerative disease groups was a reduction in VEGF (vascular endothelial growth factor) in the LBD group.
*Schulz, 2021, Movement Disorders [69]<br><br>Germany	Systematic Assessment of 10 Biomarker Candidates Focusing on α-Synuclein-Related Disorders	DLB (45)/ PD (151) / MSA (17) / PSP (38) / CBS (16) / AD (11) / FTD/ALS (15) / HC (20)	ELISA (commercial): YKL-40, Serpin A1<br>In-house assay: sTREM-2<br><br>Clinical measures: MMSE, UPDRS-III, Hoehn and Yahr	Levels of YKL-40 and sTREM2 in CSF and blood were correlated.<br>YKL-40 levels in blood and CSF were lower in the DLB group compared to AD, and no different from controls or any other neurodegenerative disease group.<br>sTREM2 levels were elevated in blood and CSF of the DLB group (and all other groups) compared to controls, but there was no difference between the neurodegenerative disease groups.<br>Serpin A1 levels were not able to be reported as the assays failed validation.
Ayton, 2022, Movement Disorders [70]<br><br>BioFINDER study *(see also above: Hall, 2018)*<br><br>Sweden	The Neuroinflammatory Acute Phase Response in Parkinsonian-Related Disorders	DLB (23) / PDD (29) / PD (155) / HC (612) / MSA (26) / PSP (22)<br><br>*NB: possible overlap in samples used from PD, PDD and HC groups with other BioFINDER cohort reports listed above.*	Mass spectrometer: inflammatory acute phase response proteins AAT, ACT, ceruloplasmin, complement C3, ferritin, fibrinogen (α, β, and γ isoforms), hemopexin, haptoglobin, and transthyretin<br>Multiplex ELISA: ferritin<br><br>Clinical measures: MMSE, UPDRS-motor, Hoehn and Yahr	Across the inflammatory acute phase response proteins tested, the degree of difference from controls was more marked in the DLB/PDD, MSA and PSP groups compared to PD.<br>In the DLB/PDD groups:<br>• AAT and ACT were elevated compared to controls.<br>• After Bonferroni correction for multiple comparisons, differences in other inflammatory proteins were not significant.<br>Inflammatory acute phase response proteins did not increase with disease severity.<br>Association of tested proteins with Parkinson's disease clinical severity measures:<br>• None identified after Bonferroni correction.<br>• Before Bonferroni correction, significant associations were found with ACT in PD, transthyretin, ferritin and α/β-fibrinogen in PDD, and α/β-fibrinogen in combined PDD/DLB group.<br>Association of tested proteins with cognition clinical severity was identified in the combined PDD/DLB group, and not in other disease groups:

Author, Year, Journal	Title	Subjects, groups	Inflammation markers assessed & methods of assessment in CSF	Outcomes/key results
				• Higher ceruloplasmin was associated with better performance on the MMSE (after Bonferroni correction)<br>• Before Bonferroni correction, a significant association was also seen with AAT, ceruloplasmin, complement C3, ferritin, fibrinogen (α, β, and γ isoforms) and hemopexin.<br>The authors conclude that the signal of elevated acute phase response proteins in PDD/DLB, and the fact that higher levels associated with better cognitive performance, support the hypothesis of a potentially protective inflammatory response early in the disease course.

Table S4. Blood

Author, Year, Journal	Title	Subjects, groups	Inflammation markers assessed & methods of assessment in blood	Outcomes/key results
*Rota, 2006, Neurological Sciences [47]<br><br>Italy	Increased intrathecal TGF-beta1, but not IL-12, IFN-γ and IL-10 levels in Alzheimer's disease patients	PDD (10) /PD (14) / AD (30) / VaD (19) / NPH (14) / HC (25)	ELISA RIA (in-house): IL-12 p40, IL-12 p70, IFN-γ, TGF-β1.<br>ELISA (commercial): IL-10, TGF-β1<br><br>Clinical measures: MMSE	No significant differences were found in the PDD group for any of the assessed markers. TGF-β1 was increased in CSF of AD cases but not serum.<br>No correlation was found between the paired measurements for each cytokine in CSF and serum.
*Ernst, 2007, J Neuroimmunology [48]<br><br>Germany	Procalcitonin is elevated in the cerebrospinal fluid of patients with dementia and acute neuroinflammation	DLB (8) / AD (40) /VaD (12) / FTD (12) / HC (50) / acute neuro-inflammation (16)	Highly sensitive commercial assay for procalcitonin [49]	Procalcitonin levels in CSF were significantly elevated in DLB (and all other dementia groups) compared to controls, whereas plasma levels were not different to controls. CSF and plasma levels of procalcitonin did not correlate in the dementia groups, but did in the acute inflammation group.
*Maetzler, 2007, Neurobiology of Disease [50]<br><br>Germany	Osteopontin is elevated in Parkinson's disease and its absence leads to reduced neurodegeneration in the MPTP model	PDD (9) / PD (21) / HC (30)	ELISA (commercial): osteopontin (same for CSF and blood)<br><br>Clinical measures: Hoehn and Yahr.	Osteopontin levels in serum and blood were significantly higher in PD/PDD compared to controls, and participants with PDD has significantly higher osteopontin in CSF, but not blood, compared to PD.
*Nielsen, 2007, Neurobiology of Disease [51] / Nielsen, 2007, Neurology [52] / Nielsen, 2012, Current Alzheimer Research [53] / Wennstrom, 2014, International Journal of Tryptophan Research [54]<br><br>Malmo Alzheimer Study<br><br>Sweden	Soluble adhesion molecules and angiotensin-converting enzyme in dementia / Plasma and CSF serpins in Alzheimer disease and dementia with Lewy bodies / Gender-dependent levels of hyaluronic acid in cerebrospinal fluid of patients with neurodegenerative dementia / Kynurenic Acid levels in cerebrospinal fluid from patients with Alzheimer's disease or dementia with Lewy bodies	DLB (39) / AD (260) / HC (34)	ELISA (commercial): sICAM-1, sVCAM-1, sPECAM-1, ACE, hyaluroinic acid (HA), MCP-1. (CSF and blood)<br>ELISA (in-house): neuroserpin (CSF only)<br><br>Rocket immunoelectrophoresis (in-house modifications): AAT, ACT (CSF and blood)<br><br>Liquid chromatography: kynurenic acid (CSF only)<br><br>Clinical measures: MMSE	Vascular inflammatory markers:<br>• Compared to controls, both dementia groups had significantly altered levels of plasma (increased sICAM-1, increased sPECAM-1) and CSF (lower sVCAM1) endothelial markers. The DLB group was distinguished from the AD group by higher sICAM-1 in plasma and CSF, and higher plasma sPECAM-1.<br>Serpins (AAT, ACT, neuroserpin; implicated in inflammation modulation):<br>• In the DLB group, worse cognition (lower MMSE scores) was associated with higher levels of AAT and t-tau in CSF.<br>• CSF serpin levels were elevated in DLB and AD groups compared to controls, and in plasma for the AD group. In models using these serpins for discrimination of DLB from AD, diagnostic performance was not better than measuring the standard diagnostic markers t-tau, p-tau and Aβ1-42<br>Hyaluroinic acid:<br>• No significant difference in levels between controls and DLB or AD groups.<br>• CSF levels of hyaluroinic acid were different between males and females in both dementia groups, and in females correlated positively with other inflammatory markers.

Author, Year, Journal	Title	Subjects, groups	Inflammation markers assessed & methods of assessment in blood	Outcomes/key results
				MCP-1 and kynurenic acid:<br>• CSF MCP-1 levels were not different between the DLB, AD and control groups. There was no effect of age or gender on levels, and MCP-1 did not correlate with kynurenic acid levels in CSF.
*Maetzler, 2009, J Neural Transmission [55]<br><br>Germany	A single-nucleotide polymorphism of the osteopontin gene may contribute to a susceptibility to Lewy body disease	DLB (9) / PDD (6) / PD (43) / HC (30)	ELISA (commercial): osteopontin (CSF and blood)<br>Commercial Primer-Extension Assay for genotyping: osteopontin allele polymorphisms SNP-66 and SNP 1239<br><br>Clinical measures: Hoehn and Yahr	Osteopontin levels were increased in CSF and blood of Lewy body disease (DLB/PDD/PD) participants compared to controls. After adjusting for age, there was no difference in osteopontin levels between DLB, PDD and PD in CSF and blood.<br>Discrimination ability of osteopontin between Lewy body disease and controls was moderate for CSF (AUC = 0.74) and poor (AUC = 0.68) for serum.<br>The SNP-66 osteopontin gene polymorphism was associated with Lewy body disease, but the genotype prevalence did not significantly correlate with osteopontin levels in CSF or blood.
Bjorkqvist, 2012, PloS one [71]<br><br>Sweden	Evaluation of a previously suggested plasma biomarker panel to identify Alzheimer's disease	DLB (37) / PDD (11) / HC (174) /AD (142) / FTD (22) / VaD (18) / depression (29)	Cytokine antibody array of 18 biomarkers (commercial): ANG-2I, CAM-1, IGFBP-6, PARC (CCL-18), PDGF-BB, RANTES, EGF, G-CSF, GDNF, IL-1 α, IL-3, IL-8, IL-11, MCP-3, M-CSF, MIP-1d, TNF-α, and TRAIL R4<br>Electrochemiluminescence assay (commercial): M-CSF, TNF-α<br><br>Clinical measures: MMSE	Attempts to validate a panel of 18 biomarkers for AD diagnosis did not find significantly different levels between DLB/PDD and AD, and the authors conclude this technique is not useful for the diagnosis of AD compared to other dementia groups.
Lanuti, 2012, Neurobiology of Aging [72]<br><br>Italy and UK	Amyloid-specific T-cells differentiate Alzheimer's disease from Lewy body dementia	DLB (14) / AD (38) / HC (22) / cerebral amyloid angiopathy (5) / inclusion body myositis (3)	Multi-color flow cytometry of T lymphocytes stimulated with Aβ1-42: markers of T cell populations (CD3, CD4, CD8), T cell effector functions (TNF-α, IFN-γ, IL-2), P-PKC-delta (Thr507), P-PKC-zeta (Thr410)<br><br>Clinical measures: MMSE, NPI, frontal assessment battery (FAB), CAF, UPDRS, Hoehn and Yahr	Characteristic T cells expressing bright levels of P-PKC-delta and P-PKC-zeta were identified in blood from AD patients and not in DLB or control groups.<br>Other markers of AD amyloid co-pathology in the DLB cohort are not reported, therefore relevance to DLB pathophysiology is difficult to determine.
Song, 2013, Internal Medicine (Japan) [73]<br><br>South Korea	Is neuroinflammation involved in the development of dementia in patients with Parkinson's disease?	PDD (45) / PD (72) / HC (84)	Commercial clinical laboratory: hsCRP and fibrinogen<br><br>Clinical measures: MMSE, CDR, CDR-SOB, Hoehn and Yahr	HsCRP and fibrinogen levels in blood were increased in PDD and PD groups compared to controls, and there was no difference between PDD and PD groups.

Author, Year, Journal	Title	Subjects, groups	Inflammation markers assessed & methods of assessment in blood	Outcomes/key results
Clough, 2015, Alzheimer Disease & Associated Disorders [74]<br><br>UK	Proinflammatory cytokines and the clinical features of dementia with Lewy bodies	DLB (18) / possible DLB (15)	Electrochemiluminescence immunoassay: IL-6, TNF-α<br><br>Clinical measures: NPI, Confusion Assessment Method (CAM; to exclude delirium), ADAS-Cog	*NB: Results from DLB and possible DLB participants were reported together.*<br>Higher IL-6 levels were correlated with poorer cognitive performance.<br>Higher TNF-α levels correlated significantly with symptoms of depression, disturbed sleep and disturbed eating.
Choi, 2016, Neurology Asia [75]<br><br>South Korea	Relationship between serum high-sensitivity C-reactive protein levels and cognitive function in patients with Parkinson's disease	PD (48) / PD-MCI (41) / PDD (24)	Latex assay, immunoturbidimetric analysis: serum hsCRP<br><br>Clinical measures: MMSE, Hoehn and Yahr, UPDRS-III	Serum hsCRP levels were higher in the PDD group compared to the non-dementia PD groups. There was no significant difference in hsCRP level between the PD-MCI group and the PD group with normal cognition.<br>HsCRP was significantly associated with disease duration and PD severity, and with performance on neuropsychological tests of visuospatial function, visual memory, and executive function after controlling for age, sex, symptom duration, education, disease severity, and UPDRS motor score on multivariate linear regression analyses.
Lue, 2016, Neurology®, Neuroimmunology & Neuroinflammation [76]<br><br>USA	Converging mediators from immune and trophic pathways to identify Parkinson disease dementia	PDD (22) / PD (52)	Multiplex antibody array: 160 proteins measured in blood, including inflammation and immune-associated molecules (i.e., IL-1β, IL-2, IL-4, IL-6, IL-10, IL-8, IL-13, IL-15, IL-17, TNF-α, TGF-β, MCP-1, VEGF, ICAM, IP-10, IFN-γ, osteopontin etc.; for full list, see: http://www.raybiotech.com/quantibody-human-cytokine-array-3000.html)<br><br>Clinical measures: MMSE, CDR, auditory verbal learning test-A7	Using an unbiased biomarker discovery approach to look for a panel of peripheral immune and inflammatory mediators that could discriminate between PDD and PD, a panel of 14 proteins was identified that, when combined with age, delivered 96% sensitivity, 89% specificity, and AUC = 0.9615.<br>These 14 proteins included chemokines (CCL 17 (TARC), CCL 22 (MDC), CXCL16), growth factors (BMP-5, FGF-4, IGF-1, NGF-R, PDGF-AA, PDGF-BB, SCF), cytokines (IL-2, IL-15, TNF-α), and vascular mediators (uPAR).
King, 2018, Journal of Neurology, Neurosurgery and Psychiatry [77] / Thomas, 2020, International Journal of Geriatric Psychiatry [78]<br><br>UK	Peripheral inflammation in prodromal Alzheimer's and Lewy body dementias / Prospective longitudinal evaluation of cytokines in mild cognitive impairment due to AD and Lewy body disease	DLB (37) / AD (20) / HC (20) /MCI-LB (38) / MCI-AD (20)	Electrochemiluminescence multiplex assay (commercial): IFN-γ, IL-10, IL-12p70, IL-13, IL-1β, IL-2, IL-4, IL-6, IL-8 and TNF-α<br>Clinical Roche cobas c702 assay: hsCRP<br><br>Clinical measures: CDR, ACE-R, UPDRS-III, Geriatric Depression Scale (GDS), ADLs, CAF<br>Amyloid PET imaging also performed	Baseline (cross-sectional) results:<br>• The MCI-LB group (and MCI-AD group) had significantly higher plasma levels of IL-10, IL-1β, IL-4 and IL-2 compared to DLB, AD and control groups. TNF-α levels were lower in MCI-LB compared to DLB.<br>• No difference was detected between LB-MCI and DLB for CRP, IFN-γ, IL-6, and IL-8.<br>• No difference in cytokines found between controls and dementia groups.

Author, Year, Journal	Title	Subjects, groups	Inflammation markers assessed & methods of assessment in blood	Outcomes/key results
			Longitudinal assessments of MCI-LB and MCI-AD participants: 12-monthly clinical assessments and blood collection up to three years. No longitudinal markers in DLB group reported	• The only significant correlation between inflammatory markers and clinical symptoms in DLB was higher IL-10 associated with greater severity of motor parkinsonism.<br>• IL-13 and IL-12p70 results were not included due to lack of detection in many samples and high inter-assay variability, respectively.<br>Longitudinal outcomes from MCI-LB and MCI-AD groups:<br>• IL-1β, IL-2, IL-4 and IL-10 were significantly lower than controls at baseline.<br>• IFN-γ, IL-1β, IL-2, IL-4, IL-6 and IL-10 decreased over time in both MCI groups, whereas IL-8 and TNF-α levels were stable. No difference in trajectory when separated into AD and LB groups.<br>• Worsening cognition correlated with decreased IFN-γ, IL-1β, IL-2, IL-4 and IL-10.<br>• No correlation was found between change in cytokine levels and parkinsonism severity.
*Surendranathan, 2018, Brain [42] / Nicastro 2020, NeuroImage - Clinical [43]<br><br>NIMROD study<br><br>UK	Early microglial activation and peripheral inflammation in dementia with Lewy bodies / Correlation of microglial activation with white matter changes in dementia with Lewy bodies	DLB (19) / HC (26)	PET: [11C]-PK11195 for TSPO (microglia)<br><br>Blood: Electrochemiluminescence immunoassay, ELISA (multiplex assay): included hsCRP, IL-2, IL-6, TNF-α, IL-12, IL-15, IFNγ, IL8, MCP-1, MIP-3a, IP10, VEGF, TNF-R1, IL-34, YKL-40 (see Table 2 in *Surendranathan et al*. for full list)<br><br>Clinical measures: MMSE, ACE-R, UPDRS-III	PET:<br>• [11C]-PK11195 binding was elevated in DLB subjects with mild disease (based on clinical cognitive scores) when compared to those with moderate/severe disease. Relative preservation of brain white matter on MRI associated with increased microglial activation, indicating that inflammation is an early event in DLB.<br>Blood inflammatory molecules:<br>• T lymphocyte-associated cytokines MIP-3a and IL-17a levels were increased, and IL-8 levels were decreased, in DLB participants compared to controls.
Villar-Pique, 2019, Journal of Neuroinflammation [79]<br><br>Germany	Plasma YKL-40 in the spectrum of neurodegenerative dementia	LBD (34) / AD (50) / HC (70) / VaD (22) / FTD (17) / CJD (78) / Neurological controls (44)	Enzyme immunoassay (commercial): YKL-40	YKL-40 was higher in LBD, and all other disease groups after correcting for age, compared to neurological controls.<br>There was no significant difference in YKL-40 levels between LBD and any other disease group.<br>Across the whole cohort, YKL-40 was associated with age, but not sex.
Amin, 2020, Journal of Neurology,	Peripheral immunophenotype in	DLB (31) / AD (31) / HC (31)	Electrochemiluminescence immunoassay (commercial): IL-1β, IL-2,	Cytokines:

Author, Year, Journal	Title	Subjects, groups	Inflammation markers assessed & methods of assessment in blood	Outcomes/key results
Neurosurgery, and Psychiatry [80]<br><br>UK	dementia with Lewy bodies and Alzheimer's disease: an observational clinical study		IL-4, IL-6, IL-8, IL-10, IL-12, IL-13, TNF-α and IFN-γ<br><br>Flow cytometry: monocytes (CD3-CD14+), T lymphocyte subsets (CD3+, CD4+ or CD8+) B lymphocytes (CD19+); activation of T or B lymphocytes (HLA-DR+); in T lymphocytes, also looked for naive cells (CD45RA+CCR7+), central memory cells (CD45RA-CCR7+), effector memory cells (CD45RA-CCR7-) and terminal effector cells (CD45RA+CCR7-)<br><br>Clinical measures: MoCA, UPDRS, NPI, CAF, FCSRT-R (free and cued selective reminding test-immediate recall), CSDD (Cornell Scale for Depression in Dementia)	• Compared to controls, the DLB group had increased frequency of IL-1β detection and increased IL-6 concentration, after correcting for age and gender.<br>• No significant difference in adjusted TNF-α and IL-10 levels between DLB and controls was detected.<br>• No correlation found between immune markers with clinical assessment tools for DLB.<br>Lymphocyte subsets:<br>• DLB participants had reduced relative numbers of CD4+ T lymphocytes and activated B lymphocytes (CD19+/HLA-DR+/MFI) compared to AD.<br>• Non-significant trend towards increased CD8+ cytotoxic T lymphocytes in DLB compared to controls and AD.
Usenko, 2020, Journal of Clinical Neuroscience [81]<br><br>Russia	Plasma cytokine profile in synucleinopathies with dementia	DLB (16) / PDD (19) / PD (29) / HC (19)	Luminex array (commercial): IFN-γ, IL4, IL6, IL-10, TNF-α, MCP-1<br><br>Clinical measures: MMSE	TNF-α, IL-6 and MCP-1 levels were elevated in DLB and PDD groups compared to controls, whereas there was no difference between the PD group and controls.<br>Compared to the PD group, TNF-α levels in DLB and PDD groups were higher, and the DLB group also had higher IFN-γ and IL-6.<br>No relationship was detected between cognitive score and the measured inflammatory markers, however whether participants were comparable in age, sex, disease duration and cognition was not reported.
Peng, 2020, International Journal of Clinical and Experimental Medicine [82]<br><br>China	Effects of butylphthalide soft capsules on cognitive function, ability of daily living, and related factors in patients with Parkinson's disease dementia	PDD (92; 46 in each treatment arm)	Immunoturbidimetry (commercial kits): CRP, cystatin C (CysC)<br><br>Clinical measures: MMSE, MoCA, UPDRS, ADLs	In this unblinded, single-centre, randomised controlled trial assessing impact of treatment with donepezil and butylphthalide soft capsules, compared to donepezil alone, inflammatory markers CRP and CysC decreased in both groups at 16 weeks and clinical outcome measures improved.<br>Levels of CRP and CysC were lower in the group that received butylphthalide soft capsules at 16 weeks.
Wang, 2020, Ageing [83]<br><br>China	Vascular, inflammatory and metabolic risk factors in relation to dementia in Parkinson's disease patients with type 2 diabetes mellitus	PDD (61; 31 with diabetes mellitus) / PD (495; 215 with diabetes mellitus) /	Immunoturbidimetric assay: hsCRP, CysC<br><br>Clinical measures: MMSE, MoCA, UPDRS, Hoehn and Yahr, NMMS (Non-	Participants with diabetes mellitus more frequently recorded elevated hsCRP (>3mg/L) levels than PDD participants.<br>CysC was identified on multiple logistic regression as a risk factor for dementia in PD patients

Author, Year, Journal	Title	Subjects, groups	Inflammation markers assessed & methods of assessment in blood	Outcomes/key results
		372 diabetes mellitus	Motor Symptoms Scale for Parkinson's Disease)	
Laguna, 2021, npj Parkinson's Disease [84]<br><br>Spain	Serum metabolic biomarkers for synucleinopathy conversion in isolated REM sleep behavior disorder	*DLB (20) / PD (15) / HC (29) / pre-DLB (15) / pre-PD (18) / iRBD-only (33)<br><br>*All study participants had polysomnographic-confirmed iRBD, of which some converted to DLB during the study.	Nuclear magnetic resonance (NMR)-based tests for protein glycosylation profiles (indicates systemic inflammatory processes.) in serum - 2D diffusion-ordered 1H NMR spectroscopy measurements<br>*NB also measured lipoproteins; total 27 parameters analysed*<br>Machine-learning approach used to develop a predictive model<br><br>*NB: only cross-sectional data is available for DLB; longitudinal data is reported for the pre-DLB group only.*	In the group of participants with iRBD who later converted to clinical DLB, the parameter *area glycB* was significantly lower in pre-DLB compared to controls who did not convert during the study (mean follow-up 10.3 ± 4.1 years).<br>Pre- and post-conversion samples were available for n=12 DLB participants, which showed higher levels of *area glycB*, and lower levels of medium LDL-P and LDL-TG post-conversion, compared to pre-conversion levels.<br>Therefore *area glycB* is proposed as a biomarker for iRBD patients who develop DLB.
Rajkumar, 2021, American Journal of Geriatric Psychiatry [85]<br><br>Norway	Next-Generation RNA-Sequencing of Serum Small Extracellular Vesicles Discovers Potential Diagnostic Biomarkers for Dementia With Lewy Bodies	DLB (10) / HC (10)	RNA profiling of serum extracellular vesicles (SEVs)<br>High throughput qPCR to verify identify DEGs<br>Ingenuity pathway analyses performed<br><br>Clinical measures: MMSE, CDR	RNA expression in SEVs from peripheral blood identified DEGs in the DLB group compared to controls, including downregulation of pro-inflammatory genes *IL1B*, *CXCL8*, and *IKBKB*, which were verified by qPCR.<br>Functional pathway and network analyses found significant enrichment of molecular pathways involving inflammation, with downregulation of proinflammatory pathways including IL-6, IL-8, IFN and T-cell receptor signalling in DLB, leading to the conclusion that immunosenescence may be an important contributor to DLB pathology.<br>Contributions from ubiquitin proteasome system dysfunction, DNA repair, and RNA post-transcriptional modification deficits were also identified.
*Schulz, 2021, Movement Disorders [69]<br><br>Germany	Systematic Assessment of 10 Biomarker Candidates Focusing on α-Synuclein-Related Disorders	DLB (45)/ PD (151) / MSA (17) / PSP (38) / CBS (16) / AD (11) / FTD/ALS (15) / HC (20)	ELISA (commercial): YKL-40, Serpin A1<br>In-house assay: sTREM-2<br><br>Clinical measures: MMSE, UPDRS-III, Hoehn and Yahr	Levels of YKL-40 and sTREM2 in CSF and blood were correlated.<br>YKL-40 levels in blood and CSF were lower in the DLB group compared to AD, and no different from controls or any other neurodegenerative disease group.<br>sTREM2 levels were elevated in blood and CSF of the DLB group (and all other groups) compared to controls, but there was no difference between the neurodegenerative disease groups.<br>Serpin A1 levels were not able to be reported as the assays failed validation.

Author, Year, Journal	Title	Subjects, groups	Inflammation markers assessed & methods of assessment in blood	Outcomes/key results
Donaghy, 2022, American Journal of Geriatric Psychiatry [86]<br><br>LewyPro, AMPLE, MIDAS, SUPErB studies<br><br>UK	Blood mRNA Expression in Alzheimer's Disease and Dementia With Lewy Bodies	DLB (38) / AD (24) / MCI-LB 55) / MCI-AD (19) / HC (28)	mRNA extracted from whole blood for RNA sequencing<br>Differentially expressed genes (DEGs) identified, gene set enrichment analysis performed<br><br>Clinical measures: ACE-R	Several DEGs were identified in the DLB group compared to controls (17 DEGs) and the AD group (18 DEGs), including when the DLB/MCI-LB and AD/MCI-AD groups were combined (2 DEGs).<br>Gene set enrichment analyses revealed:<br>• Upregulation in TNF-α signalling via NFκB pathway and the Inflammatory response pathway in DLB that did not reach statistical significance (p=0.06), but was significant in the AD group compared to controls.<br>• IFN-α and IFN-γ response pathways were upregulated in AD compared to DLB, and in MCI-AD compared to MCI-LB.
Liu, 2022, The Lancet [87]<br><br>China	QEEG indices are associated with inflammatory and metabolic risk factors in Parkinson's disease dementia: An observational study	PDD (31) / PD (47)<br><br>*NB: HC (47) group included for EEG component only.*	HsCRP, ESR, superoxide dismutase (SOD; systemic inflammatory mediator), CysC; methods not reported<br><br>Clinical measures: MMSE, UPDRS, Hoehn and Yahr	The PDD group had higher hsCRP, ESR and CysC levels compared to PD.<br>Binary logistic regression found an association between higher hsCRP and PDD, but not with PD.<br>This study also found lower levels of the metabolic marker HDL-C associated with PDD. Quantitative EEG (QEEG) differences identified in the PDD group compared to PD and controls was also correlated with plasma hsCRP (positively), SOD (positively) and HDL-C (negatively).
Oizumi, 2022, PLOS ONE [88]<br><br>Japan	Plasma sphingolipid abnormalities in neurodegenerative diseases	DLB (28) / AD (13) / HC (15)<br><br>*NB: two other cohorts included in this paper also contained PD, PSP and MSA groups compared to HC, but not compared with DLB results.*	Plasma lipidomics evaluated by liquid chromatography-tandem mass spectrometry for 324 metabolites, of which some are related to inflammation<br><br>Clinical measures: MMSE	Altered levels of ceramide sphingolipids were detected in the DLB group (and all other neurodegenerative disease groups) compared to controls. Relating to inflammation, increased levels of the astrocyte-activating molecule lactosylceramide (LacCer) and neuroinflammation-associated ceramide-1-phosphate (C1P) were detected.<br>Unique to the DLB group, lower levels of lysophospholipids were detected compared to controls. Lysophospholipids have been implicated in cell signalling and in several pro-inflammatory biological processes.[89]<br>Direct comparison of the DLB group to the AD group in the same cohort, or other cohorts reported by this paper, were not provided. It should also be noted that there were differences in age and sex distribution between the DLB and control groups.
Costantini, 2023, Immunity & Aging [90]<br><br>Italy	Different peripheral expression patterns of the nicotinic acetylcholine receptor in dementia with Lewy bodies and Alzheimer's disease	DLB (21) / AD (13) / HC (8)	RNA expression levels from PBMCs: IL-1β, IL-6, TNF-α<br><br>Clinical measures: MoCA, CDR, UPDRS-III, NPI, Mayo questionnaire for RBD, CAF	Inflammatory cytokine (IL-1β, IL-6, TNF-α) gene expression levels were higher in DLB and AD groups compared to controls.<br>Compared to AD, the DLB group had higher levels of IL-1β RNA expression, lower TNF-α and no difference was detected for IL-6 levels.

Author, Year, Journal	Title	Subjects, groups	Inflammation markers assessed & methods of assessment in blood	Outcomes/key results
				No significant associations were found between inflammatory cytokine expression, clinical disease measures or expression of nicotinic acetylcholine receptor (nAChR) subunits, although there were differences in nAChR subunit expression between study groups.

Table S5. Genetic factors

Author, Year, Journal	Title	Subjects, groups	Assessment methods	Outcomes/key results
Liu, 2016, Scientific Reports [91]<br><br>China	Lack of association between IL-10 and IL-18 gene promoter polymorphisms and Parkinson's disease with cognitive impairment in a Chinese population	PD (92) / PDD (100) / HC (473) / PD-MCI (76)	DNA genotyping: SNPs in the IL-10 promoter (rs1800871 and rs1800872) and in the IL-18 promoter (rs1946518 and rs187238)<br><br>Substrate: blood (*in vivo*)<br><br>Clinical outcomes: MMSE, UPDRS, Hoehn and Yahr, FAB, NPI, and several other cognitive assessment tools	No evidence found to support the hypothesis that IL-10 and IL-18 promoter-region SNPs are associated with cognitive impairment in PD patients of Han Chinese origin.
Conway, 2018, Molecular Neurodegeneration [92]<br><br>USA	ABI3 and PLCG2 missense variants as risk factors for neurodegenerative diseases in Caucasians and African Americans	DLB (306) / AD (2742) / PD (838) / PSP (231) / MSA (150) / HC (3351)<br><br>*NB: 67/306 DLB cases were neuro-pathologically verified*	Gene variants: ABI3_rs616338-T and PLCG2_rs72824905-G<br><br>Substrate: blood (*in vivo*)	Significant association of rare coding variants ABI3_rs616338-T (increased disease risk) and PLCG2 rs72824905-G (reduced disease risk) was only identified for the AD group, although there was a trend towards significance for the association of these variants with DLB, and not for any other groups studied.<br>PLCG2_rs72824905-G showed a suggestive association of increased risk with pathologically confirmed MSA and PSP.
*Li, 2020, International Journal of Molecular Sciences [29] / Li, 2022, Neural Regeneration Research [30]<br><br>USA	Microglia implicated in tauopathy in the striatum of neurodegenerative disease patients from genotype to phenotype / Striatal oxidative damages and neuroinflammation correlate with progression and survival of Lewy body and Alzheimer diseases	DLB (10) / PDD (8) / PD (8) /AD (27) / HC (10)	ELISA, autoradiography: TSPO for microglia; TREM2, MPO, PAR for microglia-related inflammation Genotyping (SNPs): microglia-associated genes *BIN1*, *TREM2*, *TSPO*<br><br>Substrate: postmortem brain tissue from the caudate and putamen<br><br>Clinical measures: Hoehn and Yahr stage	Significant associations were identified between biochemical markers of inflammation, SNPs, microglia, tau neuropathology, and clinical measures of disease severity and progression in LBD brains:<br>• Reduced microglia (TSPO) density and tau fibrils were observed in the Lewy body disease (DLB/PDD/PD) cohort compared with controls, and positive correlations existed between the concentration of tau fibrils and MPO.<br>• TSPO level in tissue was also haplotype dependent, with certain polymorphisms in TSPO appearing to confer increased or reduced levels.<br>• TREM2 levels increased with disease progression in the putamen of Lewy body disease brains, and there was a significant negative association between TREM2 expression and tau fibrils in DLB cases. TREM2 expression in the caudate was influenced by allele G of the *BIN1* gene, therefore this allele may be a risk factor for tauopathy.

Author, Year, Journal	Title	Subjects, groups	Assessment methods	Outcomes/key results
				• PAR concentration in the caudate of Lewy body disease brains, including PDD and DLB when analysed separately, was positively associated with disease progression.

Abbreviations

AT: α1-antitrypsin

ACE: Addenbrooke's Cognitive Examination

ACE-R: revised Addenbrooke's Cognitive Examination

ACT: α1-antichymotrypsin

AD: Alzheimer's disease

ADAS-Cog: Alzheimer's Disease Assessment Scale–Cognitive Subscale

ADLs: Activities of Daily Living

ALS: amyotrophic lateral sclerosis

aMCI: amnestic mild cognitive impairment

AUC: area under the ROC curve

BA: Brodmann Area

BIN1: bridging integrator 1

bvFTD: behavioural variant frontotemporal dementia

CAF: clinical assessment of fluctuation

CBS: corticobasal syndrome

CD: Cluster of Differentiation

CDR: Clinical Dementia Rating

CDR-SOB: Clinical Dementia Rating Sum of Boxes

CHI3L1: Chitinase-3-like protein 1 aka YKL-40

CSF: cerebrospinal fluid

CXCR4: C-X-C Motif Chemokine Receptor 4

CXCL12: C-X-C Motif Chemokine Ligand 12

CysC: cystatin C

DEG: differentially expressed genes

DLB: dementia with Lewy bodies

EIA: enzyme immune assay

ELISA: enzyme-linked immunosorbent assay

FTLD-TDP: Frontotemporal lobar degeneration with TPD-43-immunoreactive pathology

GFAP: glial fibrillary acidic protein

HA: hyaluronic acid

HADS: Hospital Anxiety and Depression Scale

HC: healthy control

HDL-C: high-density lipoprotein cholesterol

HLA-DR/DP/DQ: Human leucocyte antigen-DR-DP-DQ

hsCRP: high sensitivity C-reactive protein

IBA1: ionized calcium binding adaptor molecule 1 (aka AIF1)

IHC: immunohistochemistry

FTD-Pi: FTD-Pick's disease

GFAP: glial fibrillary acidic protein

IFN-γ: interferon-γ

IL: interleukin

iLBD: idiopathic Lewy body disease

IP-10: IFN-γ induced protein 10

iRBD: idiopathic Rapid Eye Movement (REM) sleep behaviour disorder

LBD: Lewy body dementia

LRRK2: leucine-rich repeat kinase 2

MCI: amnestic mild cognitive impairment

MCP-1: Monocyte chemoattractant protein-1

MD: muscular diseases

MHC II: major histocompatibility complex class II

MIP-3: macrophage inflammatory protein-3

MMSE: Mini-Mental State Examination

MoCA: Montreal Cognitive Assessment

MPO: myeloperoxidase

mRNA: messenger RNA

NC: neurologic controls

NFATc2: nuclear factor of activated T cells, cytoplasmic 2

NPH: normal pressure hydrocephalus

NPI: neuropsychiatric inventory

PAR: poly (ADP-Ribose)

PBMCs: peripheral blood mononuclear cells

PD: Parkinson's disease (without dementia)

PD-CIND: Parkinson's disease with cognitive impairment not dementia

PDD: Parkinson’s disease dementia

PET: positron emission tomography

PNFA: progressive non fluent aphasia

PSP: progressive supranuclear palsy

qPCR: quantitative polymerase chain reaction

RANTES: Regulated upon Activation, Normal T Cell Expressed and Presumably Secreted

RBD: REM sleep behaviour disorder

RIA: Radioimmunoassay

ROI: Regions of interest

rpDLB: rapidly progressive DLB

RT-PCR: reverse transcriptase polymerase chain reaction

SCA: spinocerebellar ataxia

sCJD: sporadic Creutzfeldt-Jakob disease

SD: semantic dementia

sICAM-1: Soluble intercellular adhesion molecule-1

SNP: single-nucleotide polymorphism

SOD: systemic inflammatory mediator

sPECAM-1: soluble platelet endothelial cell adhesion molecule-1

sTREM2: soluble TREM2

sVCAM-1: Soluble vascular adhesion molecule-1

TNF-α: tumour necrosis factor-α

TREM2: triggering receptors expressed on myeloid cell 2

TSPO: translocator protein

UPDRS: Unified Parkinson's Disease Rating Scale

VascD: Vascular dementia

YKL-40: aka Chitinase-3-like protein 1 (CHI3L1)

References

1. McGeer, P. L.; Itagaki, S.; Boyes, B. E., McGeer, E. G. Reactive microglia are positive for HLA-DR in the substantia nigra of Parkinson's and Alzheimer's disease brains. *Neurology*. 1988;*38*(8):1285-91.
2. Iseki, E.; Marui, W.; Akiyama, H.; Ueda, K., Kosaka, K. Degeneration process of Lewy bodies in the brains of patients with dementia with Lewy bodies using alpha-synuclein-immunohistochemistry. *Neuroscience letters*. 2000;*286*(1):69-73.
3. Mackenzie, I. R. Activated microglia in dementia with Lewy bodies. *Neurology*. 2000;*55*(1):132-4.
4. Rozemuller, A. J.; Eikelenboom, P.; Theeuwes, J. W.; Jansen Steur, E. N., de Vos, R. A. Activated microglial cells and complement factors are unrelated to cortical Lewy bodies. *Acta neuropathologica*. 2000;*100*(6):701-8.
5. Shepherd, C. E.; Thiel, E.; McCann, H.; Harding, A. J., Halliday, G. M. Cortical inflammation in Alzheimer disease but not dementia with Lewy bodies. *Archives of Neurology*. 2000;*57*(6):817-22.
6. Togo, T.; Iseki, E.; Marui, W.; Akiyama, H.; Ueda, K., Kosaka, K. Glial involvement in the degeneration process of Lewy body-bearing neurons and the degradation process of Lewy bodies in brains of dementia with Lewy bodies. *Journal of the neurological sciences*. 2001;*184*(1):71-5.
7. Katsuse, O.; Iseki, E., Kosaka, K. Immunohistochemical study of the expression of cytokines and nitric oxide synthases in brains of patients with dementia with Lewy bodies. *Neuropathology*. 2003;*23*(1):9-15.
8. Imamura, K.; Hishikawa, N.; Ono, K.; Suzuki, H.; Sawada, M.; Nagatsu, T.; et al. Cytokine production of activated microglia and decrease in neurotrophic factors of neurons in the hippocampus of Lewy body disease brains. *Acta Neuropathologica*. 2005;*109*(2):141-50.
9. Loeffler, D. A.; Camp, D. M., Conant, S. B. Complement activation in the Parkinson's disease substantia nigra: An immunocytochemical study. *Journal of Neuroinflammation*. 2006;*3*:29.
10. Saldana, M.; Aguilar, E.; Bonastre, M.; Marin, C.; Pujols, L.; Mullol, J.; et al. Relevance of COX-2 gene expression in dementia with Lewy bodies associated with Alzheimer pathology. *Movement Disorders*. 2008;*23*(6):804-10.
11. Castellani, R. J.; Nugent, S. L.; Morrison, A. L.; Zhu, X.; Lee, H.-g.; Harris, P. L. R.; et al. CD3 in Lewy pathology: does the abnormal recall of neurodevelopmental processes underlie Parkinson's disease. *Journal of Neural Transmission*. 2011;*118*(1):23-6.
12. Bachstetter, A. D.; Van Eldik, L. J.; Schmitt, F. A.; Neltner, J. H.; Ighodaro, E. T.; Webster, S. J.; et al. Disease-related microglia heterogeneity in the hippocampus of Alzheimer's disease, dementia with Lewy bodies, and hippocampal sclerosis of aging. *Acta Neuropathologica Communications*. 2015;*3*.
13. Streit, W. J., Xue, Q.-S. Microglia in dementia with Lewy bodies. *Brain, Behavior, and Immunity*. 2016;*55*:191-201.
14. Garcia-Esparcia, P.; Lopez-Gonzalez, I.; Grau-Rivera, O.; Garcia-Garrido, M. F.; Konetti, A.; Llorens, F.; et al. Dementia with Lewy Bodies: Molecular Pathology in the Frontal Cortex in Typical and Rapidly Progressive Forms. *Frontiers in neurology*. 2017;*8*:89.
15. Kohl, Z.; Feldewerth, J.; Hornauer, P.; Munch, M.; Winkler, J.; Schlachetzki, J. C. M.; et al. Distinct pattern of microgliosis in the olfactory bulb of neurodegenerative proteinopathies. *Neural Plasticity*. 2017;*2017*:3851262.
16. Llorens, F.; Thune, K.; Tahir, W.; Kanata, E.; Diaz-Lucena, D.; Xanthopoulos, K.; et al. YKL-40 in the brain and cerebrospinal fluid of neurodegenerative dementias. *Molecular neurodegeneration*. 2017;*12*(1):83.
17. Chong, J. R.; Chai, Y. L.; Lee, J. H.; Howlett, D.; Attems, J.; Ballard, C. G.; et al. Increased Transforming Growth Factor beta 2 in the Neocortex of Alzheimer's Disease and Dementia with Lewy Bodies is Correlated with Disease Severity and Soluble A beta(42) Load. *Journal of Alzheimers Disease*. 2017;*56*(1):157-66.
18. Wilhelmsson, U.; Andersson, D.; De Pablo, Y.; Pekny, R.; Stahlberg, A.; Mulder, J.; et al. Injury Leads to the Appearance of Cells with Characteristics of Both Microglia and Astrocytes in Mouse and Human Brain. *Cerebral Cortex*. 2017;*27*(6):3360-77.

19. Walker, D. G.; Lue, L. F.; Tang, T. M.; Adler, C. H.; Caviness, J. N.; Sabbagh, M. N.; et al. Changes in CD200 and intercellular adhesion molecule-1 (ICAM-1) levels in brains of Lewy body disorder cases are associated with amounts of Alzheimer's pathology not alpha-synuclein pathology. *Neurobiology of Aging*. 2017;*54*:175-86.
20. Ren, Q.; Ma, M.; Hashimoto, K.; Yang, J.; Hwang, S. H.; Hammock, B. D.; et al. Soluble epoxide hydrolase plays a key role in the pathogenesis of Parkinson's disease. *Proceedings of the National Academy of Sciences of the United States of America*. 2018;*115*(25):E5815-E23.
21. Erskine, D.; Ding, J.; Thomas, A. J.; Kaganovich, A.; Khundakar, A. A.; Hanson, P. S.; et al. Molecular changes in the absence of severe pathology in the pulvinar in dementia with Lewy bodies. *Movement disorders : official journal of the Movement Disorder Society*. 2018;*33*(6):982-91.
22. Santpere, G.; Garcia-Esparcia, P.; Andres-Benito, P.; Lorente-Galdos, B.; Navarro, A., Ferrer, I. Transcriptional network analysis in frontal cortex in Lewy body diseases with focus on dementia with Lewy bodies. *Brain pathology (Zurich, Switzerland)*. 2018;*28*(3):315-33.
23. Xu, J.; Sun, J.; Benzinger, T. L. S.; Perrin, R. J.; Mach, R. H.; Bales, K. R.; et al. Translocator protein in late stage Alzheimer's disease and Dementia with Lewy bodies brains. *Ann Clin Transl Neurol*. 2019;*6*(8):1423-34.
24. De Wit, N. M.; De Vries, H. E.; Den Hoedt, S.; Mulder, M. T.; Martinez-Martinez, P., Rozemuller, A. J. Astrocytic ceramide as possible indicator of neuroinflammation. *Journal of Neuroinflammation*. 2019;*16*(1):48.
25. Amin, J.; Holmes, C.; Dorey, R. B.; Tommasino, E.; Casal, Y. R.; Williams, D. M.; et al. Neuroinflammation in dementia with Lewy bodies: a human post-mortem study. *Translational Psychiatry*. 2020;*10*(1):267.
26. Iba, M.; Kim, C.; Kwon, S.; Masliah, E.; Sallin, M.; Verma, A.; et al. Neuroinflammation is associated with infiltration of T cells in Lewy body disease and alpha-synuclein transgenic models. *Journal of Neuroinflammation*. 2020;*17*(1):214.
27. Kim, C.; Beilina, A.; Smith, N.; Li, Y.; Kim, M.; Kumaran, R.; et al. LRRK2 mediates microglial neurotoxicity via NFATc2 in rodent models of synucleinopathies. *Science Translational Medicine*. 2020;*12*(565).
28. Kouli, A.; Camacho, M.; Allinson, K., Williams-Gray, C. H. Neuroinflammation and protein pathology in Parkinson's disease dementia. *Acta neuropathologica communications*. 2020;*8*(1):211.
29. Li, H.; Knight, W. C.; Yang, P.; Guo, Y.; Benzinger, T. L. S.; Xu, J.; et al. Microglia implicated in tauopathy in the striatum of neurodegenerative disease patients from genotype to phenotype. *International Journal of Molecular Sciences*. 2020;*21*(17):1-23.
30. Li, H.; Knight, W., Xu, J. Striatal oxidative damages and neuroinflammation correlate with progression and survival of Lewy body and Alzheimer diseases. *Neural Regeneration Research*. 2022;*17*(4):867-74.
31. Rajkumar, A. P.; Aarsland, D.; Bidkhori, G.; Shoaie, S.; Clarke, E.; Williams, G.; et al. Postmortem Cortical Transcriptomics of Lewy Body Dementia Reveal Mitochondrial Dysfunction and Lack of Neuroinflammation. *American Journal of Geriatric Psychiatry*. 2020;*28*(1):75-86.
32. Gate, D.; Tapp, E.; Leventhal, O.; Shahid, M.; Nonninger, T. J.; Yang, A. C.; et al. CD4(+) T cells contribute to neurodegeneration in Lewy body dementia. *Science*. 2021:eabf7266.
33. Low, C. Y. B.; Lee, J. H.; Lim, F. T. W.; Lee, C.; Ballard, C.; Francis, P. T.; et al. Isoform-specific upregulation of FynT kinase expression is associated with tauopathy and glial activation in Alzheimer's disease and Lewy body dementias. *Brain Pathol*. 2021;*31*(2):253-66.
34. Terreros-Roncal, J.; Moreno-Jimenez, E. P.; Flor-Garcia, M.; Rodriguez-Moreno, C. B.; Trinchero, M. F.; Cafini, F.; et al. Impact of neurodegenerative diseases on human adult hippocampal neurogenesis. *Science (New York, NY)*. 2021;*374*(6571):1106-13.
35. Chua, X. Y.; Chong, J. R.; Cheng, A. L.; Lee, J. H.; Ballard, C.; Aarsland, D.; et al. Elevation of inactive cleaved annexin A1 in the neocortex is associated with amyloid, inflammatory and apoptotic markers in neurodegenerative dementias. *Neurochem Int*. 2022;*152*:105251.
36. Fixemer, S.; Ameli, C.; Hammer, G.; Salamanca, L.; Huarte, O. U.; Schwartz, C.; et al. Microglia phenotypes are associated with subregional patterns of concomitant tau, amyloid-beta and alpha-synuclein pathologies in the hippocampus of patients with Alzheimer's disease and dementia with Lewy bodies. *ACTA NEUROPATHOLOGICA COMMUNICATIONS*. 2022;*10*(1).

37. Tu, H.; Zhang, Z. W.; Qiu, L.; Lin, Y.; Jiang, M.; Chia, S. Y.; et al. Increased expression of pathological markers in Parkinson's disease dementia post-mortem brains compared to dementia with Lewy bodies. *BMC Neuroscience*. 2022;*23*(1):3.
38. Iannaccone, S.; Cerami, C.; Alessio, M.; Garibotto, V.; Panzacchi, A.; Olivieri, S.; et al. In vivo microglia activation in very early dementia with Lewy bodies, comparison with Parkinson's disease. *Parkinsonism & related disorders*. 2013;*19*(1):47-52.
39. Fan, Z.; Aman, Y.; Ahmed, I.; Brooks, D. J.; Edison, P.; Chetelat, G.; et al. Influence of microglial activation on neuronal function in Alzheimer's and Parkinson's disease dementia. *Alzheimer's and Dementia*. 2015;*11*(6):608.
40. Edison, P.; Ahmed, I.; Fan, Z.; Hinz, R.; Gelosa, G.; Chaudhuri, K. R.; et al. Microglia, amyloid, and glucose metabolism in Parkinson's disease with and without dementia. *Neuropsychopharmacology*. 2013;*38*(6):938-49.
41. Femminella, G. D.; Ninan, S.; Atkinson, R.; Fan, Z.; Brooks, D. J., Edison, P. Does Microglial Activation Influence Hippocampal Volume and Neuronal Function in Alzheimer's Disease and Parkinson's Disease Dementia? *Journal of Alzheimer's disease : JAD*. 2016;*51*(4):1275-89.
42. Surendranathan, A.; Su, L.; Mak, E.; Passamonti, L.; Hong, Y. T.; Arnold, R.; et al. Early microglial activation and peripheral inflammation in dementia with Lewy bodies. *Brain*. 2018;*141*:3415-27.
43. Nicastro, N.; Mak, E.; Williams, G. B.; Surendranathan, A.; Bevan-Jones, W. R.; Passamonti, L.; et al. Correlation of microglial activation with white matter changes in dementia with Lewy bodies. *Neuroimage-Clinical*. 2020;*25*.
44. Nicastro, N.; Surendranathan, A.; Mak, E.; Rowe, J. B., O'Brien, J. T. (11) C-PK11195 PET imaging and white matter changes in Parkinson's disease dementia. *Ann Clin Transl Neurol*. 2019;*6*(10):2133-6.
45. Gomez-Tortosa, E.; Gonzalo, I.; Fanjul, S.; Sainz, M. J.; Cantarero, S.; Cemillan, C.; et al. Cerebrospinal fluid markers in dementia with lewy bodies compared with Alzheimer disease. *Archives of neurology*. 2003;*60*(9):1218-22.
46. Janssen, J. C.; Godbolt, A. K.; Ioannidis, P.; Thompson, E. J., Rossor, M. N. The prevalence of oligoclonal bands in the CSF of patients with primary neurodegenerative dementia. *Journal of neurology*. 2004;*251*(2):184-8.
47. Rota, E.; Rocca, P.; Bergamasco, B.; Ferrero, P.; Bellone, G., Emanuelli, G. Increased intrathecal TGF-beta1, but not IL-12, IFN-gamma and IL-10 levels in Alzheimer's disease patients. *Neurological Sciences*. 2006;*27*(1):33-9.
48. Ernst, A.; Morgenthaler, N. G.; Buerger, K.; Dodel, R.; Noelker, C.; Sommer, N.; et al. Procalcitonin is elevated in the cerebrospinal fluid of patients with dementia and acute neuroinflammation. *Journal of neuroimmunology*. 2007;*189*(1-2):169-74.
49. Morgenthaler, N. G.; Struck, J.; Fischer-Schulz, C., Bergmann, A. Sensitive Immunoluminometric Assay for the Detection of Procalcitonin. *Clinical Chemistry*. 2002;*48*(5):788-90.
50. Maetzler, W.; Berg, D.; Schalamberidze, N.; Gasser, T.; Melms, A.; Schott, K.; et al. Osteopontin is elevated in Parkinson's disease and its absence leads to reduced neurodegeneration in the MPTP model. *Neurobiology of Disease*. 2007;*25*(3):473-82.
51. Nielsen, H. M.; Londos, E.; Minthon, L., Janciauskiene, S. M. Soluble adhesion molecules and angiotensin-converting enzyme in dementia. *Neurobiology of Disease*. 2007;*26*(1):27-35.
52. Nielsen, H. M.; Minthon, L.; Londos, E.; Blennow, K.; Miranda, E.; Perez, J.; et al. Plasma and CSF serpins in Alzheimer disease and dementia with Lewy bodies. *Neurology*. 2007;*69*(16):1569-79.
53. Nielsen, H. M.; Palmqvist, S.; Minthon, L.; Londos, E., Wennstrom, M. Gender-dependent levels of hyaluronic acid in cerebrospinal fluid of patients with neurodegenerative dementia. *Current Alzheimer research*. 2012;*9*(3):257-66.
54. Wennstrom, M.; Nielsen, H. M.; Orhan, F.; Londos, E.; Minthon, L., Erhardt, S. Kynurenic Acid levels in cerebrospinal fluid from patients with Alzheimer's disease or dementia with lewy bodies. *International journal of tryptophan research : IJTR*. 2014;*7*:1-7.

55. Maetzler, W.; Michelis, J.; Tomiuk, J.; Melms, A.; Becker, C.; Gasser, T.; et al. A single-nucleotide polymorphism of the osteopontin gene may contribute to a susceptibility to Lewy body disease. *Journal of Neural Transmission*. 2009;*116*(5):599-605.
56. Jesse, S.; Brettschneider, J.; Sussmuth, S. D.; Landwehrmeyer, B. G.; Von Arnim, C. A. F.; Ludolph, A. C.; et al. Summary of cerebrospinal fluid routine parameters in neurodegenerative diseases. *Journal of Neurology*. 2011;*258*(6):1034-41.
57. Wang, Y.; Hancock, A. M.; Bradner, J.; Chung, K. A.; Quinn, J. F.; Peskind, E. R.; et al. Complement 3 and factor h in human cerebrospinal fluid in Parkinson's disease, Alzheimer's disease, and multiple-system atrophy. *The American journal of pathology*. 2011;*178*(4):1509-16.
58. Wennstrom, M.; Surova, Y.; Hall, S.; Nilsson, C.; Minthon, L.; Hansson, O.; et al. The Inflammatory Marker YKL-40 Is Elevated in Cerebrospinal Fluid from Patients with Alzheimer's but Not Parkinson's Disease or Dementia with Lewy Bodies. *PloS one*. 2015;*10*(8):e0135458.
59. Wennstrom, M.; Hall, S.; Nagga, K.; Londos, E.; Minthon, L., Hansson, O. Cerebrospinal fluid levels of IL-6 are decreased and correlate with cognitive status in DLB patients. *Alzheimers Res Ther*. 2015;*7*(1):63.
60. Janelidze, S.; Hertze, J.; Santillo, A.; Hansson, O.; Blennow, K.; Zetterberg, H.; et al. Cerebrospinal fluid neurogranin and YKL-40 as biomarkers of Alzheimer's disease. *Ann Clin Transl Neurol*. 2016;*3*(1):12-20.
61. Del Campo, M.; Galimberti, D.; Elias, N.; Boonkamp, L.; Pijnenburg, Y. A.; van Swieten, J. C.; et al. Novel CSF biomarkers to discriminate FTLD and its pathological subtypes. *Ann Clin Transl Neurol*. 2018;*5*(10):1163-75.
62. Hall, S.; Janelidze, S.; Surova, Y.; Widner, H.; Zetterberg, H., Hansson, O. Cerebrospinal fluid concentrations of inflammatory markers in Parkinson’s disease and atypical parkinsonian disorders. *Scientific Reports*. 2018;*8*(1):13276.
63. Janelidze, S.; Francardo, V.; Cenci, M. A.; Lindqvist, D.; Hall, S.; Londos, E.; et al. Increased CSF biomarkers of angiogenesis in Parkinson disease. *Neurology*. 2015;*85*(21):1834-42.
64. Lindqvist, D.; Hall, S.; Surova, Y.; Nielsen, H. M.; Janelidze, S.; Brundin, L.; et al. Cerebrospinal fluid inflammatory markers in Parkinson's disease--associations with depression, fatigue, and cognitive impairment. *Brain, behavior, and immunity*. 2013;*33*:183-9.
65. Paterson, R. W.; Slattery, C. F.; Poole, T.; Nicholas, J. M.; Magdalinou, N. K.; Toombs, J.; et al. Cerebrospinal fluid in the differential diagnosis of Alzheimer's disease: clinical utility of an extended panel of biomarkers in a specialist cognitive clinic. *Alzheimer's research & therapy*. 2018;*10*(1):32.
66. Hu, W. T.; Howell, J. C.; Ozturk, T.; Gangishetti, U.; Kollhoff, A. L.; Hatcher-Martin, J. M.; et al. CSF Cytokines in Aging, Multiple Sclerosis, and Dementia. *Frontiers in immunology*. 2019;*10*:480.
67. Morenas-Rodríguez, E.; Alcolea, D.; Suárez-Calvet, M.; Muñoz-Llahuna, L.; Vilaplana, E.; Sala, I.; et al. Different pattern of CSF glial markers between dementia with Lewy bodies and Alzheimer’s disease. *Scientific Reports*. 2019;*9*(1):7803.
68. Lourenco, M. V.; Ribeiro, F. C.; Santos, L. E.; Beckman, D.; Melo, H. M.; Sudo, F. K.; et al. Cerebrospinal fluid neurotransmitters, cytokines, and chemokines in Alzheimer's and Lewy body diseases. *Journal of Alzheimer's Disease*. 2021;*82*(3):1067-74.
69. Schulz, I.; Kruse, N.; Gera, R. G.; Kremer, T.; Cedarbaum, J.; Barbour, R.; et al. Systematic Assessment of 10 Biomarker Candidates Focusing on α-Synuclein-Related Disorders. *Movement Disorders*. 2021;*36*(12):2874-87.
70. Ayton, S.; Hall, S.; Janelidze, S.; Kalinowski, P.; Palmqvist, S.; Belaidi, A. A.; et al. The Neuroinflammatory Acute Phase Response in Parkinsonian-Related Disorders. *Mov Disord*. 2022;*37*(5):993-1003.
71. Bjorkqvist, M.; Ohlsson, M.; Minthon, L., Hansson, O. Evaluation of a previously suggested plasma biomarker panel to identify Alzheimer's disease. *PLoS ONE*. 2012;*7*(1):e29868.
72. Lanuti, P.; Ciccocioppo, F.; Bonanni, L.; Marchisio, M.; Lachmann, R.; Tabet, N.; et al. Amyloid-specific T-cells differentiate Alzheimer's disease from Lewy body dementia. *Neurobiology of aging*. 2012;*33*(11):2599-611.

73. Song, I.-U.; Kim, Y.-D.; Cho, H.-J., Chung, S.-W. Is neuroinflammation involved in the development of dementia in patients with Parkinson's disease? *Internal Medicine*. 2013;*52*(16):1787-92.
74. Clough, Z.; Jeyapaul, P.; Zotova, E., Holmes, C. Proinflammatory cytokines and the clinical features of dementia with lewy bodies. *Alzheimer Dis Assoc Disord*. 2015;*29*(1):97-9.
75. Choi, S.-M.; Kim, B. C.; Kang, K. W.; Choi, K.-H.; Nam, T.-S.; Kim, J.-T.; et al. Relationship between serum high-sensitivity C-reactive protein levels and cognitive function in patients with Parkinson's disease. *Neurology Asia*. 2016;*21*(4):349-56.
76. Lue, L. F.; Schmitz, C. T.; Snyder, N. L.; Chen, K. W.; Walker, D. G.; Davis, K. J.; et al. Converging mediators from immune and trophic pathways to identify Parkinson disease dementia. *Neurology-Neuroimmunology & Neuroinflammation*. 2016;*3*(1).
77. King, E.; O'Brien, J. T.; Donaghy, P.; Morris, C.; Barnett, N.; Olsen, K.; et al. Peripheral inflammation in prodromal Alzheimer's and Lewy body dementias. *J Neurol Neurosurg Psychiatry*. 2018;*89*(4):339-45.
78. Thomas, A. J.; Hamilton, C. A.; Donaghy, P. C.; Martin-Ruiz, C.; Morris, C. M.; Barnett, N.; et al. Prospective longitudinal evaluation of cytokines in mild cognitive impairment due to AD and Lewy body disease. *Int J Geriatr Psychiatry*. 2020;*35*(10):1250-9.
79. Villar-Pique, A.; Schmitz, M.; Hermann, P.; Goebel, S.; Bunck, T.; Varges, D.; et al. Plasma YKL-40 in the spectrum of neurodegenerative dementia. *Journal of Neuroinflammation*. 2019;*16*.
80. Amin, J.; Boche, D.; Clough, Z.; Teeling, J.; Williams, A.; Gao, Y.; et al. Peripheral immunophenotype in dementia with Lewy bodies and Alzheimer's disease: An observational clinical study. *Journal of Neurology, Neurosurgery & Psychiatry*. 2020;*91*(11):1219-26.
81. Usenko, T. S.; Nikolaev, M. A.; Miliukhina, I. V.; Bezrukova, A. I.; Senkevich, K. A.; Gomzyakova, N. A.; et al. Plasma cytokine profile in synucleinophaties with dementia. *Journal of Clinical Neuroscience*. 2020;*78*:323-6.
82. Peng, S., Huang, Y. Effects of butylphthalide soft capsules on cognitive function, ability of daily living, and related factors in patients with parkinson's disease dementia. *International Journal of Clinical and Experimental Medicine*. 2020;*13*(10):7758-65.
83. Wang, Q. Vascular, inflammatory and metabolic risk factors in relation to dementia in parkinson's disease patients with type 2 diabetes mellitus. *Movement Disorders*. 2020;*35*(SUPPL 1):S190.
84. Laguna, A.; Xicoy, H.; Tolosa, E.; Serradell, M.; Vilas, D.; Gaig, C.; et al. Serum metabolic biomarkers for synucleinopathy conversion in isolated REM sleep behavior disorder. *NPJ Parkinson's disease*. 2021;*7*(1):40.
85. Rajkumar, A. P.; Hye, A.; Lange, J.; Manesh, Y. R.; Ballard, C.; Fladby, T.; et al. Next-Generation RNA-Sequencing of Serum Small Extracellular Vesicles Discovers Potential Diagnostic Biomarkers for Dementia With Lewy Bodies. *The American journal of geriatric psychiatry : official journal of the American Association for Geriatric Psychiatry*. 2021;*29*(6):573-84.
86. Donaghy, P. C.; Cockell, S. J.; Martin-Ruiz, C.; Coxhead, J.; Kane, J.; Erskine, D.; et al. Blood mRNA Expression in Alzheimer's Disease and Dementia With Lewy Bodies. *Am J Geriatr Psychiatry*. 2022;*30*(9):964-75.
87. Liu, H.; Deng, B.; Zhou, H.; Wu, Z.; Chen, Y.; Weng, G.; et al. QEEG indices are associated with inflammatory and metabolic risk factors in Parkinson's disease dementia: An observational study. *eClinicalMedicine*. 2022;*52*:101615.
88. Oizumi, H.; Sugimura, Y.; Totsune, T.; Kawasaki, I.; Ohshiro, S.; Baba, T.; et al. Plasma sphingolipid abnormalities in neurodegenerative diseases. *PLoS ONE*. 2022;*17*(12 December):e0279315.
89. Tan, S. T.; Ramesh, T.; Toh, X. R., Nguyen, L. N. Emerging roles of lysophospholipids in health and disease. *Progress in Lipid Research*. 2020;*80*:101068.
90. Costantini, E.; Carrarini, C.; Borrelli, P.; De Rosa, M.; Calisi, D.; Consoli, S.; et al. Different peripheral expression patterns of the nicotinic acetylcholine receptor in dementia with Lewy bodies and Alzheimer's disease. *Immunity and Ageing*. 2023;*20*(1):3.

91. Liu, Z.; Guo, J.; Wang, Y.; Li, K.; Kang, J.; Wei, Y.; et al. Lack of association between IL-10 and IL-18 gene promoter polymorphisms and Parkinson's disease with cognitive impairment in a Chinese population. *Scientific reports*. 2016;*6*:19021.
92. Conway, O. J.; Carrasquillo, M. M.; Wang, X.; Bredenberg, J. M.; Reddy, J. S.; Strickland, S. L.; et al. ABI3 and PLCG2 missense variants as risk factors for neurodegenerative diseases in Caucasians and African Americans. *Molecular neurodegeneration*. 2018;*13*(1):53.
